# Spatial layout planning of cable based on a hybrid method combining interference obstacle local post-insertion and improved Dubins curves

**DOI:** 10.1371/journal.pone.0359351

**Published:** 2026-10-05

**Authors:** Xiaoyang Zhang, Zhigang Xu, Songkai Liu, Bing Cai, Bo Deng, Chao Kang

**Affiliations:** 1 State Key Laboratory of Robotics and Intelligent Systems, Shenyang Institute of Automation, Chinese Academy of Sciences, Shenyang, China; 2 University of Chinese Academy of Sciences, Beijing, China; 3 School of Mechanical Engineering, Jiangsu University of Science and Technology, Zhenjiang, China; Southwest Jiaotong University, CHINA

## Abstract

Cables in three-dimensional (3D) wall-adherent routing must simultaneously satisfy terminal direction vectors and bending curvature constraints, making forward design and precise pre-cutting a significant challenge. To address this, this paper proposes a hybrid planning method, termed IOLPDC, which combines interference obstacle local post-insertion with improved Dubins curves. The approach first utilizes an undirected graph spanning tree to unfold the 3D routing environment into a two-dimensional (2D) plane. Within this 2D domain, a multi-waypoint Dubins curve is introduced, and a post-insertion strategy is developed. This strategy generates a highly efficient Dubins path by initially ignoring obstacles, followed by binary-tree detouring, pruning, and redundant waypoint elimination to achieve a high-quality feasible path satisfying both directional and curvature constraints. Additionally, for local non-wall-adherent regions arising during 3D reconstruction, an engineering transition solver based on a 3D Dubins path is implemented via a Differential Evolution algorithm. Simulation results in a planar environment demonstrate that compared with Hybrid A* and RRT*Dubins, the proposed IOLPDC method reduces path length by 13.23% and 8.51%, and shortens median planning time by 50.00% and 34.38%, respectively. Experimental verification confirms that cables pre-cut according to the planned lengths precisely match the actual routed paths, effectively eliminating the traditional “cut-to-fit” process and demonstrating strong engineering practicality.

## 1 Introduction

Cable path planning is a pivotal stage in the development of complex systems, such as aerospace vehicles, transportation equipment, and high-end mechatronic systems. The quality of cable routing directly governs assembly efficiency, structural reliability, and long-term operational safety; furthermore, it serves as a decisive factor affecting overall system performance [1–3]. In complex spatial environments, such as equipment cabins with densely arranged structures and narrow channels, traditional manual routing proves inefficient and inconsistent, failing to meet the rigorous demands of intelligent assembly and forward design [4,5]. Consequently, automated cable path planning under diverse engineering constraints has emerged as a critical research priority in computer-aided design and intelligent manufacturing.

Early methodologies primarily relied on classical graph search algorithms, such as the Lee, Dijkstra, and A* algorithms [6–9]. While these methods provide intuitive and deterministic solutions for geometric shortest paths, they focus predominantly on path length while neglecting the essential curvature constraints required in engineering. To bridge this gap, many studies have adopted a two-stage strategy: initially generating an obstacle-avoiding polyline path and subsequently smoothing it via post-processing techniques, such as B-spline fitting or fillet rounding, to satisfy minimum bending radius requirements [10,11]. For instance, Kim et al. utilized B-spline optimization following a JPS-Theta search to generate natural routing shapes, while Qu et al. employed ant colony optimization for 3D branch pipelines with added fillets [12,13]. However, these two-stage strategies exhibit a fundamental limitation: post-processing smoothing can cause the trajectory to deviate from the original plan, potentially leading to collisions with previously avoided obstacles. This issue is particularly pronounced when the bending radius constraint is large relative to the available free space.

To overcome the limitations of post-processing methods for curvature constraints, the field of mobile robot path planning has developed a series of methods that directly incorporate curvature constraints into the planning process, providing useful algorithmic references for cable routing. These include Hybrid A* with non-holonomic extensions, RRT*Dubins using Dubins curves as connection primitives, and the KBQ-RRT algorithm that achieves smooth motion planning in complex constrained environments through dual-tree optimization [14–18]. Dang et al. [19] introduced improved analytic expansions and motion primitive fine-tuning in Hybrid A*, effectively reducing collision risk and improving path smoothness for non-holonomic robots. Ye et al. [20] employed bidirectional tree optimization and motion primitive expansion in KBQ-RRT* to achieve smooth motion planning in complex environments. Although these curvature-constrained path planning methods have been successful in mobile robot scenarios, their direct application to cable routing faces fundamental challenges. When obstacles are dense, and the space is too narrow, Dubins paths may become infeasible [21]. RRT-based methods, due to random sampling, produce path solutions with high variance and many redundant bends, which significantly increase the need for clamps, saddles, and assembly costs; and Hybrid A* suffers from a sharp drop in search efficiency in cluttered environments.

Beyond curvature constraints, cable routing must also satisfy wall-adherent requirements to reduce vibration and prevent interference with moving parts [22,23]. Hermansson et al. [24] used a Cosserat rod model and a contact potential energy field to make flexible harnesses automatically adhere to structural surfaces during assembly motion. Yang et al. [25] proposed an A*-ant colony hybrid algorithm that forces paths to be generated on equipment surfaces through wall-adherence penalty terms, clearance constraints, and surface mesh search spaces. Reducing the 3D wall-adherent routing problem to a 2D plane is also a feasible strategy. Rambeaud and Zeghloul [26] proposed a shortest path planning algorithm on polyhedral surfaces based on an unfolding strategy, whose core idea is to unfold the 3D polyhedral surface into a 2D plane, thus converting the 3D path planning problem into a classic 2D shortest path problem. Li et al. [27] used a similar method to unfold the developable structural surfaces inside complex aerospace cabins uniformly into a 2D plane. However, existing methods usually focus only on the smoothness of paths in the 2D unfolded plane and lack a systematic framework for managing local “out-of-plane” bending transitions that arise during the reconstruction of 2D paths into 3D space, particularly for cases where the start/end direction vectors of the cable are not coplanar with the laying surface.

Based on the preceding analysis, cable path planning faces dual challenges: first, the simultaneous satisfaction of terminal orientation vectors and minimum bending radii. Second, the lack of systematic methods for addressing out-of-plane direction vectors during wall-adherent routing. To address these issues, this paper proposes a hybrid method combining interference obstacle local post-insertion and improved Dubins curves (IOLPDC). The primary innovations are summarized as follows:

(1) A local post-insertion strategy for interfering obstacles is proposed. An initial baseline Dubins path is first generated ignoring obstacles, then binary-tree detouring, pruning, and redundant point elimination are performed for interfering obstacles. This approach avoids path distortion caused by post-processing smoothing from the outset, shifting the paradigm from the traditional “obstacle-avoidance-then-smoothing” sequence to a curvature-native planning approach, ensuring the exact reproducibility of path length in actual laying.
(2) An undirected graph spanning tree is used to reduce the 3D wall-adherent environment to a 2D plane. For local cable segments where the start/end direction vectors are not coplanar with the laying surface when restoring the 2D routing path to the 3D space, a differential evolution algorithm is used to solve a 3D Dubins engineering transition path for corresponding local post-processing, ensuring the engineering feasibility of the planned cable path.

The structure of this paper is as follows: Section 2 describes the planar unfolding method for the 3D wall-adherent cable routing space. Section 3 details methods about the IOLPDC algorithm framework. Section 4 validates the method through algorithm comparison and experiments. Section 5 concludes the paper and discusses future work.

## 2 Representation of 2D unfolding environments for 3D cables space

To facilitate spatial cable routing, a planar unfolding strategy is adopted to convert the 3D spatial problem into a more tractable 2D equivalent. The process entails performing path planning in the 2D domain, prior to an inverse coordinate transformation for final 3D layout reconstruction. This method leverages undirected graph spanning trees to model the 3D routing environment, incorporating recursive backtracking algorithms to generate all feasible 2D planar representations.

### 2.1 3D cables space description based on an undirected graph spanning tree

During cable routing within 3D environments, the routing area is typically subject to defined spatial constraints. Conventionally, the routing space is modeled as a bounded 3D rectangular volume, with the initial and terminal points of the cable assumed to be located on its boundary surfaces. Adhering to the wall-adherence principle, the routing space is first unfolded into a 2D plane to facilitate path planning. This study employs a methodology based on undirected graph spanning trees to achieve 3D-to-2D space conversion, systematically enumerating all potential boundary surfaces traversed by the cable. To elucidate the conversion principle: an undirected graph G=⟨V,E⟩ is defined, where V represents a non-empty set of vertices and E denotes the set of edges as unordered pairs connecting these vertices. Consider the routing spaces illustrated in Fig 1, which features six and eight boundary surfaces, respectively. Under the wall-adherence constraint, cable paths are restricted to these surfaces. A trajectory initiates on a starting surface, traverses a sequence of adjacent surfaces, and terminates on a target surface. Since different surface traversal sequences yield distinct planar unfolding configurations, the boundary surfaces are abstracted as vertices. By excluding the top surface—reflecting the practical constraints of cable ducts—and traversing all connected paths, the corresponding undirected graphs and adjacency matrices are generated as follows:

**Fig 1:**
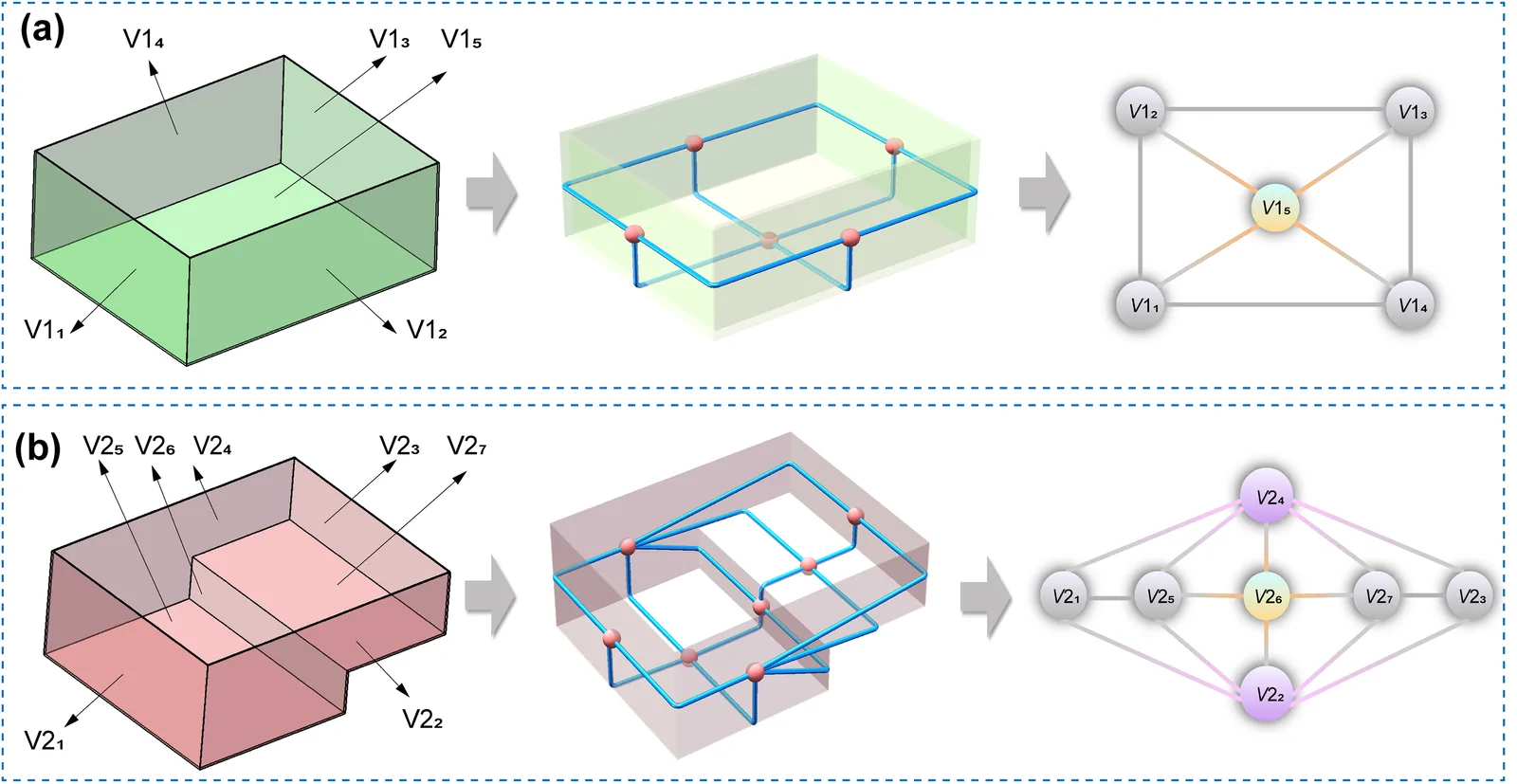
Abstracting 3D cable environments into undirected graphs, with vertices representing boundary surfaces and edges denoting adjacency: (a) 6-surface environment $G_{1}$; (b) 8-surface environment $G_{2}$.


$$\begin{array}{c} A_{\text{1}}=[\begin{array}{ccccc} \text{0} & \text{1} & \text{0} & \text{1} & \text{1}\\ \text{1} & \text{0} & \text{1} & \text{0} & \text{1}\\ \text{0} & \text{1} & \text{0} & \text{1} & \text{1}\\ \text{1} & \text{0} & \text{1} & \text{0} & \text{1}\\ \text{1} & \text{1} & \text{1} & \text{1} & \text{0} \end{array}] \end{array}$$ (1)

$$\begin{array}{c} A_{\text{2}}=[\begin{array}{ccccccc} \text{0} & \text{1} & \text{0} & \text{1} & \text{1} & \text{0} & \text{0}\\ \text{1} & \text{0} & \text{1} & \text{0} & \text{1} & \text{1} & \text{1}\\ \text{0} & \text{1} & \text{0} & \text{1} & \text{0} & \text{0} & \text{1}\\ \text{1} & \text{0} & \text{1} & \text{0} & \text{1} & \text{1} & \text{1}\\ \text{1} & \text{1} & \text{0} & \text{1} & \text{0} & \text{1} & \text{0}\\ \text{0} & \text{1} & \text{0} & \text{1} & \text{1} & \text{0} & \text{1}\\ \text{0} & \text{1} & \text{1} & \text{1} & \text{0} & \text{1} & \text{0} \end{array}] \end{array}$$ (2)

where the element $a_{ij}$ in row i and column j of the adjacency matrix represents the topological relationship between boundary surfaces i and j. If the surfaces are adjacent, $a_{ij}=\;\text{1}$; otherwise, $a_{ij}=\;\text{0}$.

### 2.2 2D expanded environment representation of 3D cable space

Section 2.1 establishes the methodology for 3D-to-2D spatial unfolding. However, the adjacency matrix typically reveals multiple feasible unfolding sequences, necessitating a systematic analysis to identify and select valid configurations. In this study, a recursive depth-first search (DFS) algorithm is implemented to exhaustively identify all potential unfolding patterns.

For the 6-surface configuration in Fig 1(a), assuming the initial cable position is on surface ${v\text{1}}_{\text{1}}$ and the terminal point is on surface ${v\text{1}}_{\text{3}}$, all feasible paths of the wall-mounted laid cable passing through the wiring surfaces can be listed as follows:


$$\begin{array}{c} {P\text{1}}_{\text{Vertex}}=\{\begin{array}{c} {v\text{1}}_{\text{1}}\to {v\text{1}}_{\text{2}}\to {v\text{1}}_{\text{3}}\\ {v\text{1}}_{\text{1}}\to {v\text{1}}_{\text{4}}\to {v\text{1}}_{\text{3}}\\ {v\text{1}}_{\text{1}}\to {v\text{1}}_{\text{5}}\to {v\text{1}}_{\text{3}}\\ \;{v\text{1}}_{\text{1}}\to {v\text{1}}_{\text{2}}\to {v\text{1}}_{\text{5}}\to {v\text{1}}_{\text{3}}\\ {v\text{1}}_{\text{1}}\to {v\text{1}}_{\text{4}}\to {v\text{1}}_{\text{5}}\to {v\text{1}}_{\text{3}}\\ {v\text{1}}_{\text{1}}\to {v\text{1}}_{\text{5}}\to {v\text{1}}_{\text{2}}\to {v\text{1}}_{\text{3}}\\ {v\text{1}}_{\text{1}}\to {v\text{1}}_{\text{5}}\to {v\text{1}}_{\text{4}}\to {v\text{1}}_{\text{3}}\\ {v\text{1}}_{\text{1}}\to {v\text{1}}_{\text{2}}\to {v\text{1}}_{\text{5}}\to {v\text{1}}_{\text{4}}\to {v\text{1}}_{\text{3}}\\ {v\text{1}}_{\text{1}}\to {v\text{1}}_{\text{4}}\to {v\text{1}}_{\text{5}}\to {v\text{1}}_{\text{2}}\to {v\text{1}}_{\text{3}} \end{array}\} \end{array}$$ (3)

Similarly, for the 8-surface scenario depicted in Fig 1(b), assuming a start surface ${v\text{2}}_{\text{5}}$ and a target surface ${v\text{2}}_{\text{7}}$, all feasible paths of the wall-mounted laid cable passing through the wiring surfaces can be determined as follows:


$$\begin{array}{c} {P\text{2}}_{\text{Vertex}}=\{\begin{array}{c} {v\text{2}}_{\text{5}}\to {v\text{2}}_{\text{2}}\to {v\text{2}}_{\text{7}}\\ {v\text{2}}_{\text{5}}\to {v\text{2}}_{\text{4}}\to {v\text{2}}_{\text{7}}\\ {v\text{2}}_{\text{5}}\to {v\text{2}}_{\text{6}}\to {v\text{2}}_{\text{7}}\\ \;{v\text{2}}_{\text{5}}\to {v\text{2}}_{\text{1}}\to {v\text{2}}_{\text{2}}\to {v\text{2}}_{\text{7}}\\ {v\text{2}}_{\text{5}}\to {v\text{2}}_{\text{1}}\to {v\text{2}}_{\text{4}}\to {v\text{2}}_{\text{7}}\\ {v\text{2}}_{\text{5}}\to {v\text{2}}_{\text{2}}\to {v\text{2}}_{\text{3}}\to {v\text{2}}_{\text{7}}\\ {v\text{2}}_{\text{5}}\to {v\text{2}}_{\text{2}}\to {v\text{2}}_{\text{6}}\to {v\text{2}}_{\text{7}}\\ \ldots \\ {v\text{2}}_{\text{5}}\to {v\text{2}}_{\text{6}}\to {v\text{2}}_{\text{4}}\to {v\text{2}}_{\text{1}}\to {v\text{2}}_{\text{2}}\to {v\text{2}}_{\text{3}}\to {v\text{2}}_{\text{7}} \end{array}\} \end{array}$$ (4)

As the geometric complexity of the routing environment increases, the number of feasible paths between the initial and terminal surfaces grows substantially. For instance, Case $G_{\text{2}}$ yields 45 distinct unfolding paths. From an engineering perspective, computational focus is primarily directed toward unfolding configurations that involve fewer surface transitions, as these typically represent more efficient routing candidates.

### 2.3 Space coordinate transformation

After determining the sequence of traversed surfaces within the 3D routing environment, a spatial coordinate transformation is executed to unfold the surfaces onto a 2D plane. This study adapts the Denavit-Hartenberg (D-H) parameter convention—originally developed for robotic joint-link transformations—to model the unfolding process [28]. The unfolding of the routing space is conceptualized as a kinematic chain consisting of multiple rigid bodies undergoing rotational motion around common edges. Specifically, the shared edges between adjacent surfaces are treated as revolute joint axes. Local reference frames are established on each surface, with each traversed face serving as a geometric “link” within the kinematic chain. Following the D-H convention, the reference frame for each surface is systematically defined. Finally, by precisely adjusting the joint angles (θ) between consecutive links, all boundary surfaces are co-planarized onto a unified 2D coordinate system.

## 3 Methods

### 3.1 Ethics statements

**Ethical approval:** All methodologies and used data comply with standards for academic integrity, ensuring no ethical conflicts throughout this research.

### 3.2 Generation method for multi-waypoint dubins curves

By transforming the spatial problem into a planar one, cable layout is converted into a 2D path planning task. Given that cables are subject to curvature constraints, the planned trajectory must maintain $C^{\text{1}}$ continuity (smoothness). To address this, this paper adopts the Dubins curve concept for multi-waypoint scenarios. As established by Dubins, the shortest planar path between two directed points under a maximum curvature constraint consists of maximum-curvature circular arcs and straight-line segments [29].

For planar cable planning, the layout area is denoted as G(x,y). The routing requires the determination of the allowable bending radius r and vector parameters for the initial and terminal points, $P_{s}\left(x_{s},y_{s},\theta_{s}\right)$ and $P_{t}\left(x_{t},y_{t},\theta_{t}\right)$. Based on the initial and terminal positions defined as $P_{\text{0}}(\text{1}\text{0},\text{1}\text{0},\text{0})$ and $P_{\text{1}}(\text{5}\text{0},\text{5}\text{0},\text{0})$, six feasible Dubins path patterns—LSL, LSR, RSL, RSR, RLR, and LRL—are generated and illustrated in Fig 2. The optimal curve is selected by identifying the pattern with the minimum length.

**Fig 2:**
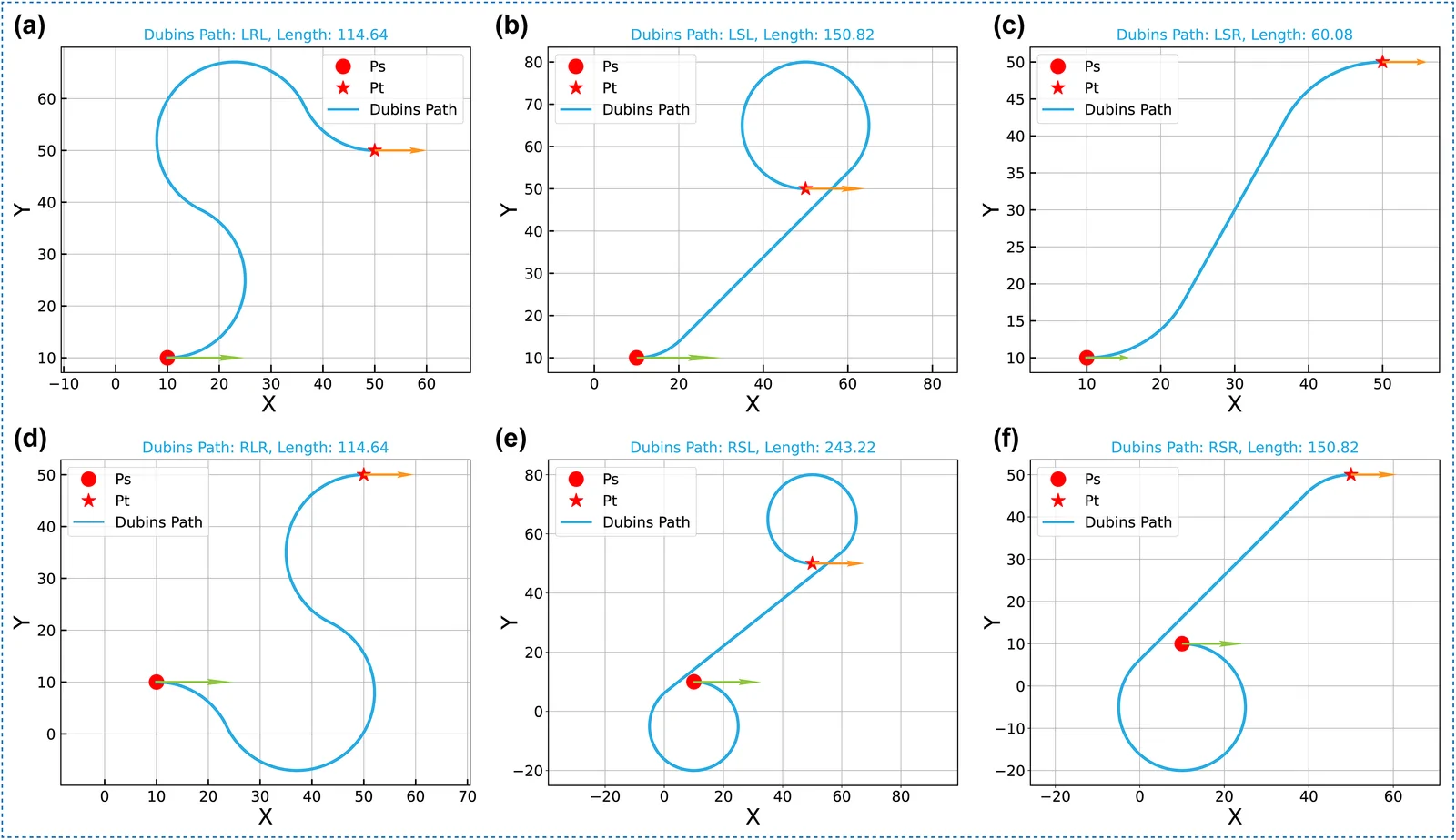
Six fundamental Dubins path patterns under *C*^1^ continuity constraints: (a) LRL, (b) LSL, (c) LSR, (d) RLR, (e) RSL, (f) RSR.

Standard Dubins paths are primarily suited for shortest-path searches in obstacle-free environments. However, since actual cable environments contain various obstacles, the path must incorporate a series of waypoints to facilitate detouring. Consequently, a multi-waypoint Dubins curve planning method is adopted. Assume there are n waypoints $P_{\text{1}},P_{\text{2}},\ldots ,P_{i},\ldots ,P_{n}$ between the start point S and terminal point T (Fig 3). For each waypoint $P_{i}\left(x_{i},y_{i},\theta_{i}\right)$, the coordinates $\left(x_{i},y_{i}\right)$ are fixed, while the orientation $\theta_{i}\;$remains a free variable. The total path is decomposed into n+1 Dubins sub-paths, where $P_{i}$ serves as the terminal point of the i-th sub-path and the starting point of the (i+1)-th sub-path. By iteratively optimizing the orientation set $\{\theta_{i}\}$ to effectively reduce the cumulative length of all sub-paths, a high-quality feasible multi-waypoint Dubins trajectory is obtained.

**Fig 3:**
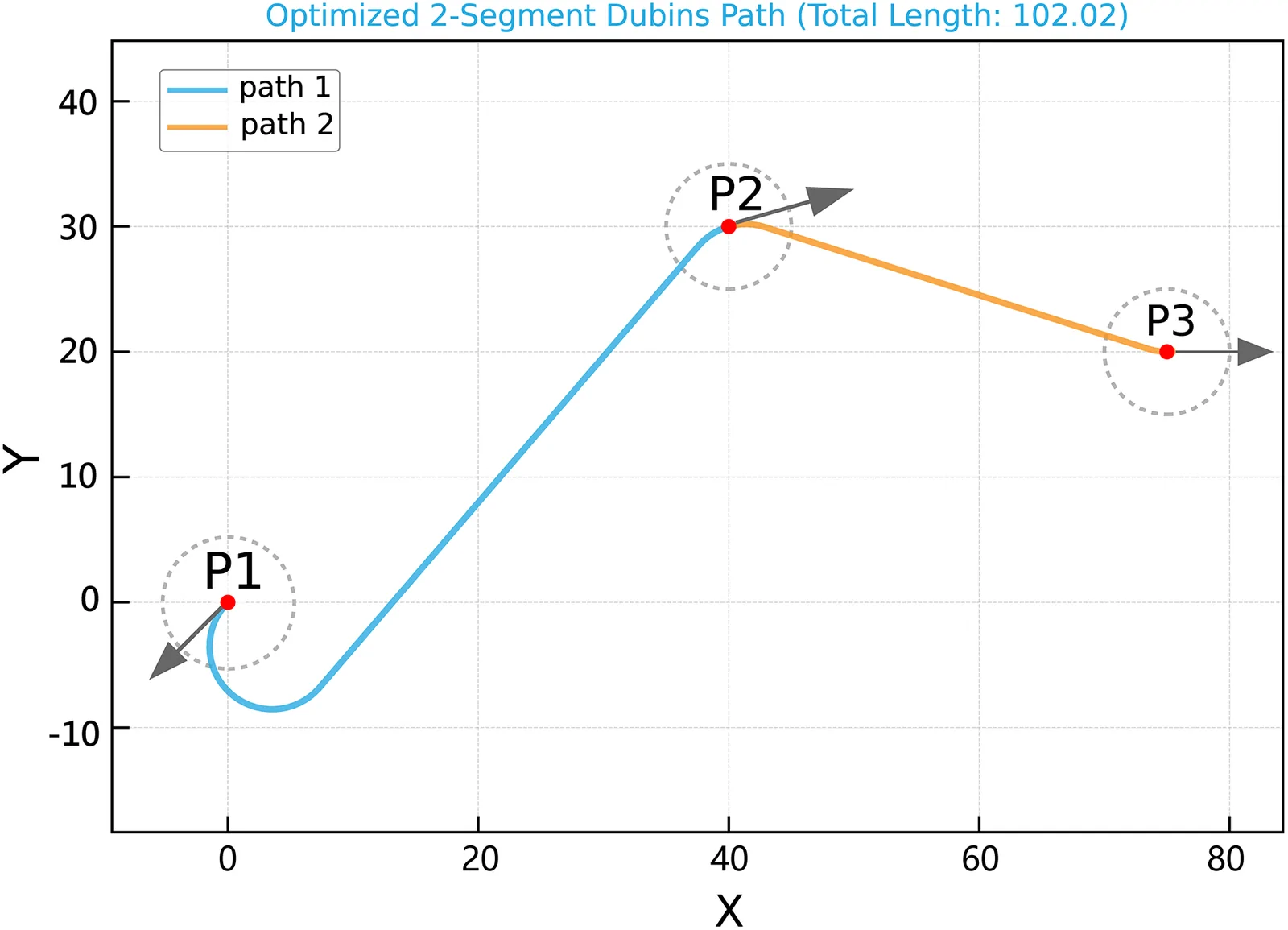
Multi-waypoint Dubins curve planning.

For n intermediary points, the optimization involves n free variables $\theta_{i}$. As n increases, the problem evolves into a high-dimensional non-convex optimization challenge with potential local optima. Gradient-based methods are often unsuitable due to the discontinuous nature of the objective function, particularly when Dubins pattern types switch (e.g., from LSL to RSR). Therefore, this study employs a Particle Swarm Optimization (PSO) algorithm to perform a parallel search across multiple orientation combinations.

The PSO algorithm explores the solution space for a high-quality near-optimal solution through information sharing and collaboration among particles in the solution space. In this context, while waypoint coordinates are fixed, their corresponding routing directions are iteratively adjusted. The objective function and the kinematic update equations for the particles are defined as follows:

Variable: $X=\left(\theta_{\text{1}},\theta_{\text{2}},\ldots ,\theta_{i},\ldots ,\theta_{n}\right)$

Objective function:


$$\begin{array}{c} f\left(\theta_{\text{1}},\theta_{\text{2}},\ldots ,\theta_{n}\right)=\text{min}\sum_{i=\text{1}}^{n+\text{1}}L_{\text{Dubins}}\left(P_{i-\text{1}},\theta_{i-\text{1}},P_{i},\theta_{i}\right) \end{array}$$ (5)

Constrain conditions:$r=r_{min},\theta_{i}\in [\text{0},\text{2}\pi )$

Velocity update:


$$\begin{array}{c} v_{i}^{t+\text{1}}=\omega v_{i}^{t}+c_{\text{1}}r_{\text{1}}\left(pbest_{i}^{t}-x_{i}^{t}\right)+c_{\text{2}}r_{\text{2}}\left(gbest^{t}-x_{i}^{t}\right) \end{array}$$ (6)

Position update:


$$\begin{array}{c} x_{i}^{t+\text{1}}=x_{i}^{t}+v_{i}^{t+\text{1}} \end{array}$$ (7)

where $L_{\text{Dubins}}$ denotes the length of a sub-path, $v_{i}^{t}$ and $x_{i}^{t}$ represent the velocity and position of particle i at iteration t, and pbest and gbest are the current historical local and global optimal solutions found by the particles, respectively. The adaptive inertia weight ω is defined to balance exploration and exploitation:


$$\begin{array}{c} \omega =\omega_{min}+\left(\omega_{max}-\omega_{min}\right)\frac{maxgen-t}{maxgen} \end{array}$$ (8)

where maxgen is the maximum number of generations.

### 3.3 Path generation via interference obstacle local post-insertion

Building upon the multi-waypoint Dubins curve generation method discussed previously, this section proposes a novel path generation strategy characterized by the local post-insertion of interfering obstacles. The algorithm initiates by generating an initial baseline Dubins path between the initial and terminal points, momentarily disregarding all obstacles. Subsequently, the path is checked for interference with obstacles in a sequential manner. Upon detecting a collision, the algorithm identifies detour waypoints to modify the trajectory. Once an obstacle-free path is established, a redundancy check is performed iteratively to eliminate unnecessary waypoints. Finally, by traversing all potential detour branches, the most efficient feasible path is selected as the final trajectory. The comprehensive workflow is depicted in Fig 4 and executed through the following structured steps:

**Fig 4:**
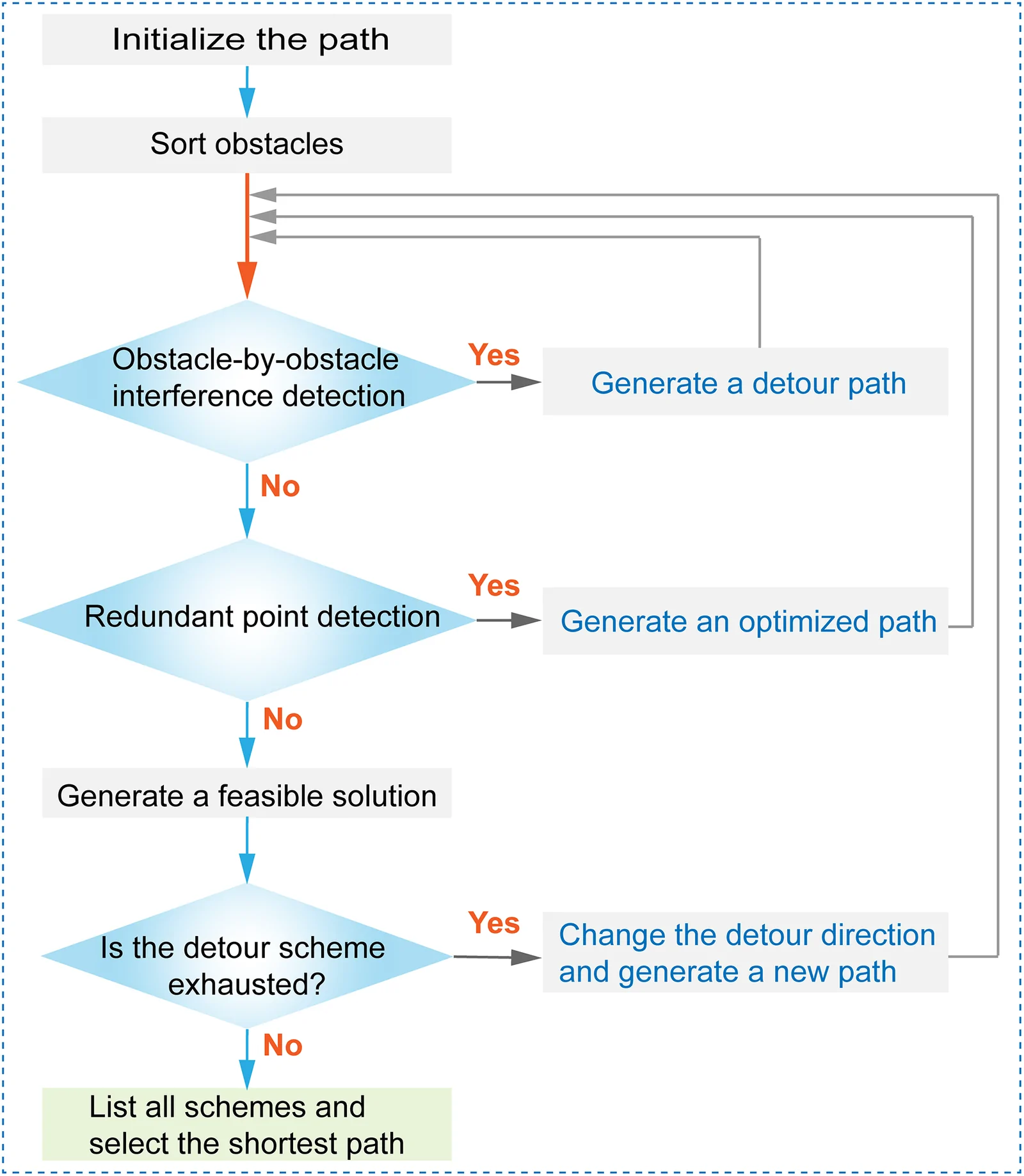
Path generation strategy of interference obstacle local post-insertion.

**Step 1:** Generate an initial baseline Dubins path between the start and goal points, ignoring all environmental obstacles.
**Step 2:** Sort Obstacles.
**Step 3:** The algorithm evaluates the intersection between the current path and each obstacle following the predefined sorting order. If interference is detected, the obstacle is treated as a detour node within a binary-tree structure. A branch direction is then selected to synthesize a detour trajectory using improved Dubins curves. This interference detection and path update cycle is performed recursively until the resulting trajectory is entirely collision-free.
**Step 4:** Iteratively attempt to remove each waypoint and verify whether the resulting path interferes with the obstacle associated with the removed waypoint. If no interference occurs, the waypoint is deemed redundant and deleted; subsequently, the waypoint sequence is updated to generate a refined Dubins path, followed by a return to Step 3.
**Step 5:** If no redundant waypoints are detected in Step 4, the current path is output as a feasible solution.
**Step 6:** Explore alternative detour directions around obstacles and regenerate candidate paths to expand the solution space.
**Step 7:** Collect all branch paths and select the shortest solution that satisfies the curvature constraints among all feasible candidates.

The algorithmic progression is schematically illustrated in Fig 5. As shown in Fig 5(a), the process initiates with a baseline trajectory that disregards all obstacles. Following the obstacle sorting phase (Fig 5(b)), the algorithm detects an intersection between the path and obstacle No. 2 (Fig 5(c)). A detour path is then generated by inserting waypoints, and the refined trajectory is re-evaluated for interference against all obstacles. Once a collision-free path is achieved—marking the completion of Step 3 (Fig 5(d))—the redundancy check begins. When it is detected that removing the waypoint associated with obstacle No. 2 does not result in interference with said obstacle (Fig 5(e)), the waypoint is deemed redundant and eliminated. Steps 3 and 4 are repeated iteratively until the path is refined into a feasible solution (Fig 5(f)). Subsequently, the algorithm explores alternative detour directions (Fig 5(g)) to ensure that the final output represents an efficient and high-quality path satisfying all engineering constraints (Fig 5(h)).

**Fig 5:**
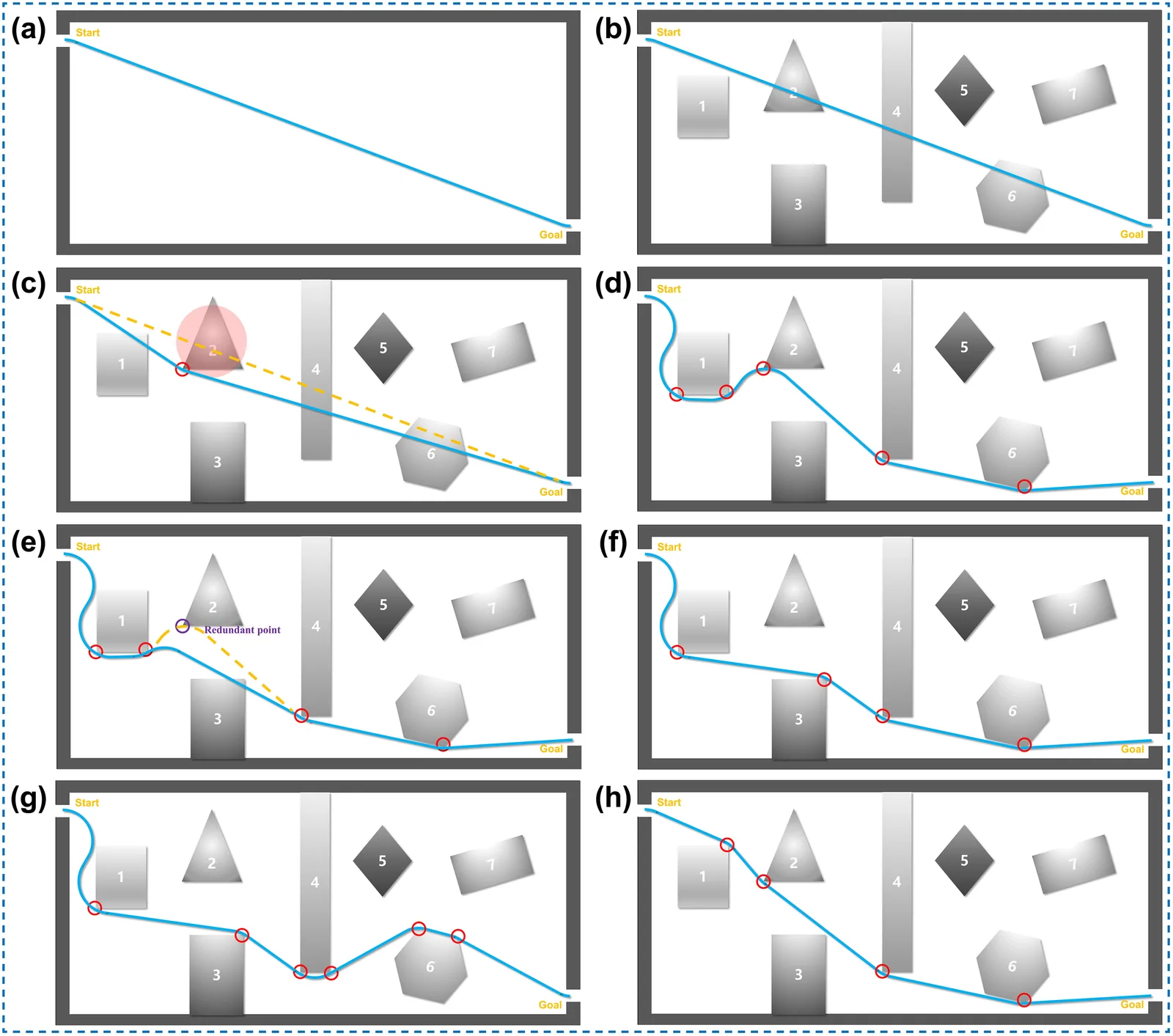
Schematic diagram of path generation strategy: (a) Step 1, (b) Step 2, (c)& (d) Step 3, (e) Step 4, (f) Step 5, (g) Step 6, (h) Step 7.

#### 3.3.1 Obstacle sorting.

As obstacles are post-inserted into the path sequentially, a deterministic insertion order must be established. This study adopts a sorting method based on a proximity coefficient $W_{i}$, calculated for each obstacle as:


$$\begin{array}{c} W_{i}=T_{i}-S_{i} \end{array}$$ (9)

where $T_{i}$ represents the Euclidean distance from the obstacle center to the cable terminal point, and $S_{i}$ denotes the distance from the obstacle center to the cable start point. Obstacles are then sorted in descending order of $W_{i}$. As illustrated in Fig 5(b), this ordering follows the intuitive progression of cable laying from the starting position to the destination and serves to maintain a consistent processing sequence during path updates.

#### 3.3.2 Obstacle interference detection.

A critical component of the proposed method is the accurate determination of path-obstacle interference. Given the 2D nature of the unfolded environment and the multi-segment structure of the improved Dubins path, the trajectory is decomposed into its constituent linear segments and circular arcs. Interference detection is then performed independently for each geometric primitive against the polygonal obstacles.

In cable routing and related engineering domains, such as piping and equipment layout, approximating obstacles as polygons or bounding boxes is a standard simplification strategy to balance computational complexity with algorithmic feasibility [21,27]. Accordingly, in the proposed framework, obstacles are modeled as polygons to facilitate efficient interference detection while maintaining high fidelity to real-world geometric constraints.

(a) **Interference Detection between Line Segments and Obstacles**

As obstacles are modeled as polygons, detecting interference between the cable path and obstacles reduces to a line segment intersection problem in a 2D plane. To optimize computational efficiency while maintaining precision, a hybrid approach combining the Bounding Box Test (BBT) and the Crossing Test is employed.

The BBT rapidly excludes non-intersecting segment pairs by checking their orthogonal projections. If the projections of two segments onto the x or y axes do not overlap, they cannot intersect. The projection boundaries for a line segment i are defined as:


$$\begin{array}{c} \left\{ \begin{array}{c} @rx_{\text{1}}^{min}=min\left(x_{p\text{1}},x_{p\text{2}}\right),\\ x_{\text{1}}^{max}=max\left(x_{p\text{1}},x_{p\text{2}}\right),\\ y_{\text{1}}^{min}=min\left(y_{p\text{1}},y_{p\text{2}}\right),\\ y_{\text{1}}^{max}=max\left(y_{p\text{1}},y_{p\text{2}}\right), \end{array}\right. \end{array}$$ (10)

where $\left(x_{p\text{1}},y_{p\text{1}}\right)$ and $\left(x_{p\text{2}},y_{p\text{2}}\right)$ are the endpoints of segment i. Intersection is conclusively ruled out if any of the following conditions are met:


$$\begin{array}{c} x_{\text{1},max}<x_{\text{2},min}\vee x_{\text{2},max}<x_{\text{1},min}\vee y_{\text{1},max}<y_{\text{2},min}\vee y_{\text{2},max}<y_{\text{1},min} \end{array}$$ (11)

If the BBT is inconclusive, the Crossing Test is performed using vector cross products to determine the positional relationship between segments AB and CD. For endpoints $A\left(A_{x},A_{y}\right)$, $B\left(B_{x},B_{y}\right)$, $C\left(C_{x},C_{y}\right)$, and $D\left(D_{x},D_{y}\right)$, the 2D cross product is:


$$\begin{array}{c} \text{cross2D}(A,B,C)=\left(B_{x}-A_{x}\right)\left(C_{y}-A_{y}\right)-\left(B_{y}-A_{y}\right)\left(C_{x}-A_{x}\right) \end{array}$$ (12)

Two segments intersect if and only if the endpoints of each segment lie on opposite sides of the line containing the other segment, expressed as:


$$\begin{array}{c} \left\{ \begin{array}{c} @rcross\text{2}D(A,B,C)\cdot cross\text{2}D(A,B,D)<\text{0}\\ cross\text{2}D(C,D,A)\cdot cross\text{2}D(C,D,B)<\text{0} \end{array}\right. \end{array}$$ (13)

(b) **Interference detection between circular arcs and obstacles**

For a circular arc segment, the algorithm first calculates the minimum distance $d_{min}$om the arc center to each edge of the polygonal obstacle. If $d_{min}>r$here r is the arc radius), no interference exists. If $d_{min}\leq r$ detailed intersection check is executed.

The intersection between a polygonal edge $E_{i}=\left(V_{i},V_{i+\text{1}}\right)$ and the circle is modeled parametrically:


$$\begin{array}{c} \left\{ \begin{array}{c} x(t)=x_{\text{1}}+t\Delta x\\ y(t)=y_{\text{1}}+t\Delta y \end{array}\right.\;\;\;,\;t\in [\text{0},\text{1}] \end{array}$$ (14)

Substituting these into the circle equation ${\left(x-O_{x}\right)}^{\text{2}}+{\left(y-O_{y}\right)}^{\text{2}}=r^{\text{2}}$ yields the quadratic form $at^{\text{2}}+bt+c=\text{0}$, with coefficients:


$$\begin{array}{c} \left\{ \begin{array}{c} @la=\Delta x^{\text{2}}+\Delta y^{\text{2}}\\ b=\text{2}\left[\Delta x\left(x_{\text{1}}-O_{X}\right)+\Delta y\left(y_{\text{1}}-O_{y}\right)\right]\\ c={\left(x_{\text{1}}-O_{x}\right)}^{\text{2}}+{\left(y_{\text{1}}-O_{y}\right)}^{\text{2}}-r^{\text{2}} \end{array}\right. \end{array}$$ (15)

For real roots t∈[0,1], the intersection point Q is identified. Its angular position α relative to the center O is then computed as $\alpha =\text{atan2}\left(Q_{y}-O_{y},Q_{x}-O_{x}\right)$. Interference is confirmed if α falls within the arc’s angular domain $\left[\theta_{\text{1}},\theta_{\text{2}}\right]$:

If *a* lies within the arc’s angular domain, the intersection can be judged by:


$$\begin{array}{c} \left\{ \begin{array}{c} @r\theta_{\text{1}}\leq a\leq \theta_{\text{2}}\;if\;\theta_{\text{1}}\;\leq \theta_{\text{2}}\\ a\in [\theta_{\text{1}},\text{2}\pi ]\cup [\text{0},\theta_{\text{2}}]\;if\;\theta_{\text{1}}\;>\theta_{\text{2}} \end{array}\right. \end{array}$$ (16)

#### 3.3.3 Interference obstacle avoidance path search.

(1) Determination of Obstacle Vertex Direction

Upon detecting interference between a Dubins path and a polygonal obstacle, specific vertices must be identified as detour waypoints. The algorithm first extracts the obstacle’s vertices and computes the signed area of the resulting polygon to determine the vertex traversal direction. A positive signed area indicates a counter-clockwise (CCW) traversal, while a negative value indicates a clockwise (CW) direction. The signed area, A, is calculated as:


$$\begin{array}{c} A=\frac{\text{1}}{\text{2}}\sum_{i=\text{0}}^{m-\text{1}}\left(x_{i}y_{i+\text{1}}-x_{i+\text{1}}y_{i}\right) \end{array}$$ (17)

where $\left(x_{i},y_{i}\right)$ represents the coordinates of the i-th vertex, and m is the total number of vertices.

In practical scenarios, obstacles may possess both convex and concave vertices. To facilitate waypoint selection, convex hull processing is performed to approximate the obstacle as a convex polygon. For a vertex set $P=\{P_{\text{1}},P_{\text{2}},\ldots ,P_{n}\}$, the convexity of any vertex $P_{i}$ is determined by the sign of the cross product of its adjacent edge vectors [30]. A positive result identifies a convex vertex, while a non-positive result indicates a concave one. By excluding concave points, the set V of convex vertices is obtained (Fig 6). It is important to note that this convex hull is utilized solely for identifying potential detour waypoints and does not alter the obstacle’s original geometry; thus, subsequent path iterations may still traverse the original concave regions if permissible.

**Fig 6:**
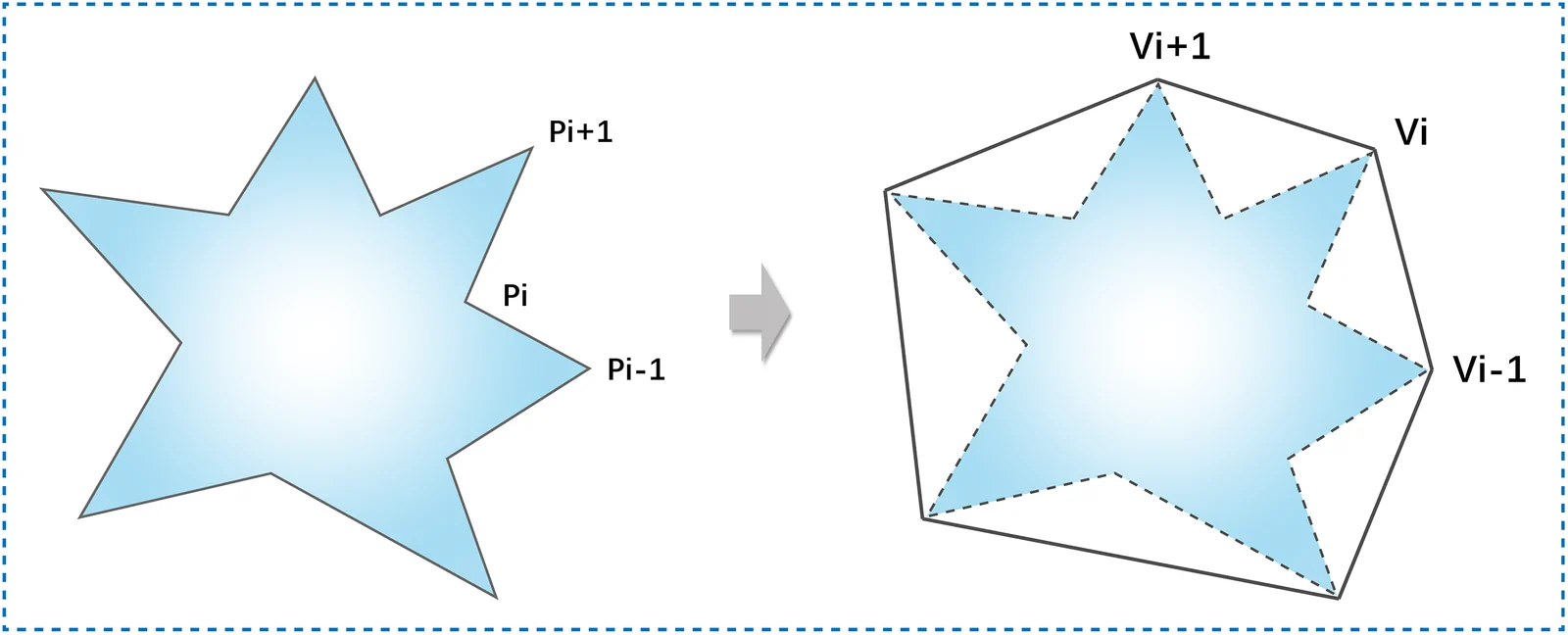
Convex hull processing for concave-convex obstacles.

(2) Waypoint Selection Methodology

When a path segment intersects an obstacle, new detour trajectories are synthesized using an improved Dubins curve algorithm and intermediate waypoints. Let V denote the set of convex vertices ordered counter-clockwise. For any vertex $V_{i}\in V$, its adjacent vertices are defined as $A=V_{i-\text{1}}$ and $B=V_{i+\text{1}}$ (Figs 7(a) and 7(b)).

**Fig 7:**
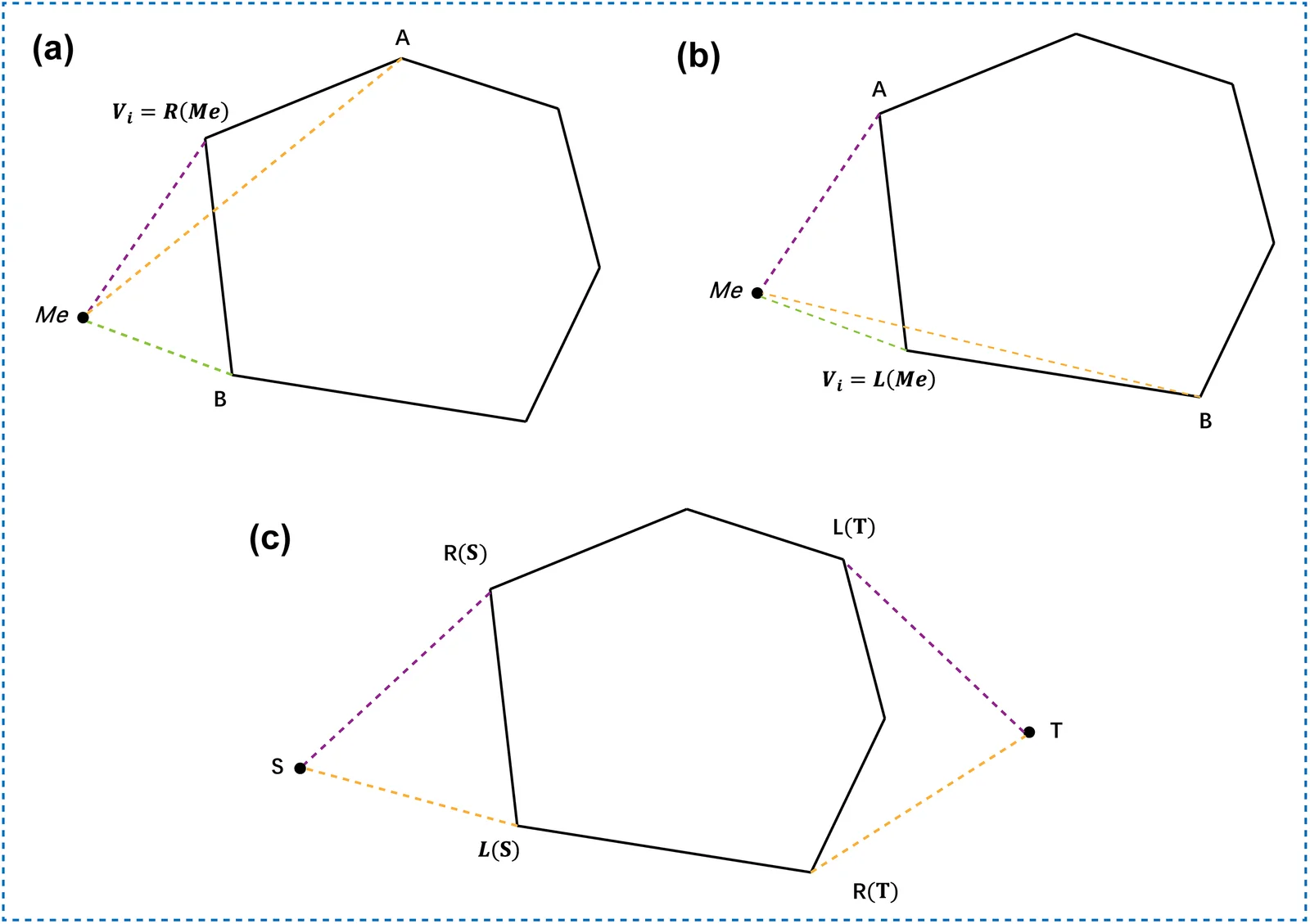
Waypoint determination: (a) Left tangent points; (b) Right tangent points; (c) Two detour paths for the obstacle.

Assuming $M_{e}$ is an external starting point, the left and right tangent points on the polygon are determined based on their geometric properties. The left tangent point, $L\left(M_{e}\right)$, is identified when:


$$\begin{array}{c} \left(M_{e}V_{i}\times M_{e}A\right)\geq \text{0}\;\text{and}\;\left(M_{e}V_{i}\times M_{e}B\right)\geq \text{0} \end{array}$$ (18)

Similarly, the right tangent point, $R\left(M_{e}\right)$, satisfies:


$$\begin{array}{c} \left(M_{e}V_{i}\times M_{e}A\right)\leq \text{0}\;\text{and}\;\left(M_{e}V_{i}\times M_{e}B\right)\leq \text{0} \end{array}$$ (19)

where × denotes the 2D cross product.

To bypass an obstacle between two exterior points S(start) and T (terminal), the algorithm identifies the corresponding tangent points L(S),R(S),L(T), and R(T). Consequently, two potential detour candidates are generated (Fig 7(c)):

Left Detour Path: Traverses the sequence S→L(S)→R(T)→T.
Right Detour Path: Traverses the sequence S→R(S)→L(T)→T.

If L(S)=R(T) or R(S)=L(T), the tangent points coincide, and the detour simplifies to a trajectory with a single intermediary waypoint.

#### 3.3.4 Algorithm complexity analysis.

Under the proposed local post-insertion strategy, let N denote the total number of obstacles involved in the final detour paths. The number of vertices per convex polygon obstacle is assumed to be a constant, m. In the PSO algorithm, P represents the population size, I denotes the maximum number of iterations, and the maximum number of waypoints in a single path is of the same order as N, denoted as O(N).

**Space Complexity:** The algorithm maintains all explored binary-tree branches and their associated trajectory data. Each branch stores a sequence of waypoints, each defined by planar coordinates and an orientation angle; thus, the maximum storage required per path is O(N). For a total of N obstacles requiring detours, the worst-case number of branch paths is ${\text{2}}^{N}$, leading to an overall space complexity of $O\left({\text{2}}^{N}\cdot N\right)$. In practical scenarios, if K obstacles are situated on the environment boundary, the complexity is reduced to $O\left({\text{2}}^{N-K}\cdot N\right)$.

**Time complexity:** For a single optimization of an N-waypoint problem using PSO, the worst-case time complexity is O(P·I·N). For path search involving N waypoints, from sequential obstacle insertion to sequential redundant point detection, assuming a total of 2N PSO optimizations, the worst-case time complexity for a single feasible path solution is $O\left(\text{2}P\cdot I\cdot N^{\text{2}}\right)$. For K obstacles located on the environment boundary, the maximum number of binary-tree branches is ${\text{2}}^{N-K}$, so the worst-case time complexity of the complete binary-tree search is:


$$\begin{array}{c} T(N)=O\left({\text{2}}^{N-K+\text{1}}\cdot P\cdot I\cdot N^{\text{2}}\right) \end{array}$$ (20)

It should be noted that the computational complexity of each interference detection and tangent point search is O(m) (where m is the number of obstacle vertices), which is negligible compared to the cost of the PSO optimization for improved Dubins paths; therefore, the above complexity analysis mainly reflects the dominant costs of PSO and post-insertion iterations. Although the expression grows exponentially, in practice the pruning mechanism significantly reduces the number of effective branches, and N is the number of obstacles actually involved in all detour paths, which is usually much smaller than the total number of global obstacles M. Moreover, the complexity of this algorithm is independent of the overall environment size, allowing it to complete planning within acceptable time for practical cable layout scenarios.

### 3.4 Cable bending processing from planar path to 3D spatial environment

#### 3.4.1 Problem description.

The preceding sections provide a solution for cable routing where endpoints lie within the wall-adjacent plane. However, in practical installation, terminal orientation vectors may not align with the laying surface. In such instances, localized path treatment is required to adjust the cable orientation, transitioning the path back into the plane to maintain compatibility with the aforementioned routing procedures.

**Case 1: Internal bending process for adjacent folding planes**

As described in Section 2, after completion of in-plane routing, when the routing environment is folded and restored to 3D space, bending treatment of cables emerging between adjacent folding planes becomes necessary. As shown in Fig 8, the transition point *P*_*AB*_ within the plane requires bending processing. Based on the geometric relationships, a smooth transition at the bend can be readily achieved by applying the minimum bending radius *R*; for the sake of brevity, details are omitted for brevity.

**Fig 8:**
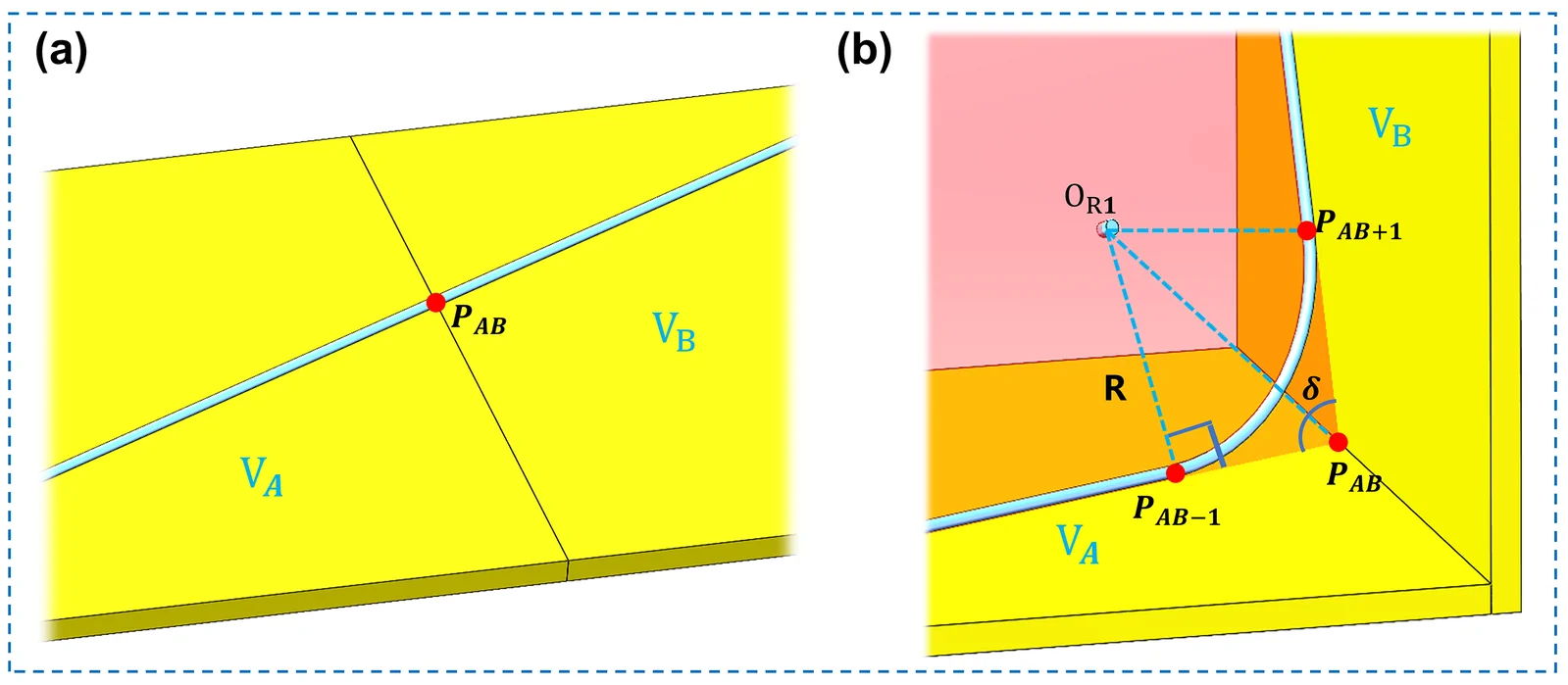
Case 1: (a) 2D Path Planning; (b) Bending treatment.

**Case 2: External bending processing for adjacent folding planes**

As shown in Fig 9, the cable path following external bending cannot strictly adhere to the wall within the local region of the planar folding zone due to the minimum bending radius R constraint. To address this, an engineering processing method is implemented: during the cable transition from plane $V_{C}$ to $V_{D}$ while maintaining the original routing path on $V_{C}$, the intersection $P_{CD}$ between the folding line and the cable is defined as the starting point for local processing, with its direction vector remaining consistent with the original cable orientation. By extending along the original wall-adhering path for a distance of 3R, the endpoint $P_{CD+\text{1}}$ of the local treatment path is determined, with its direction vector aligned to the spatial orientation following the folding transition; subsequently, a 3D Dubins engineering transition solver, as detailed in Section 3.3.2, is employed to resolve the non-wall-adherent segment.

**Fig 9:**
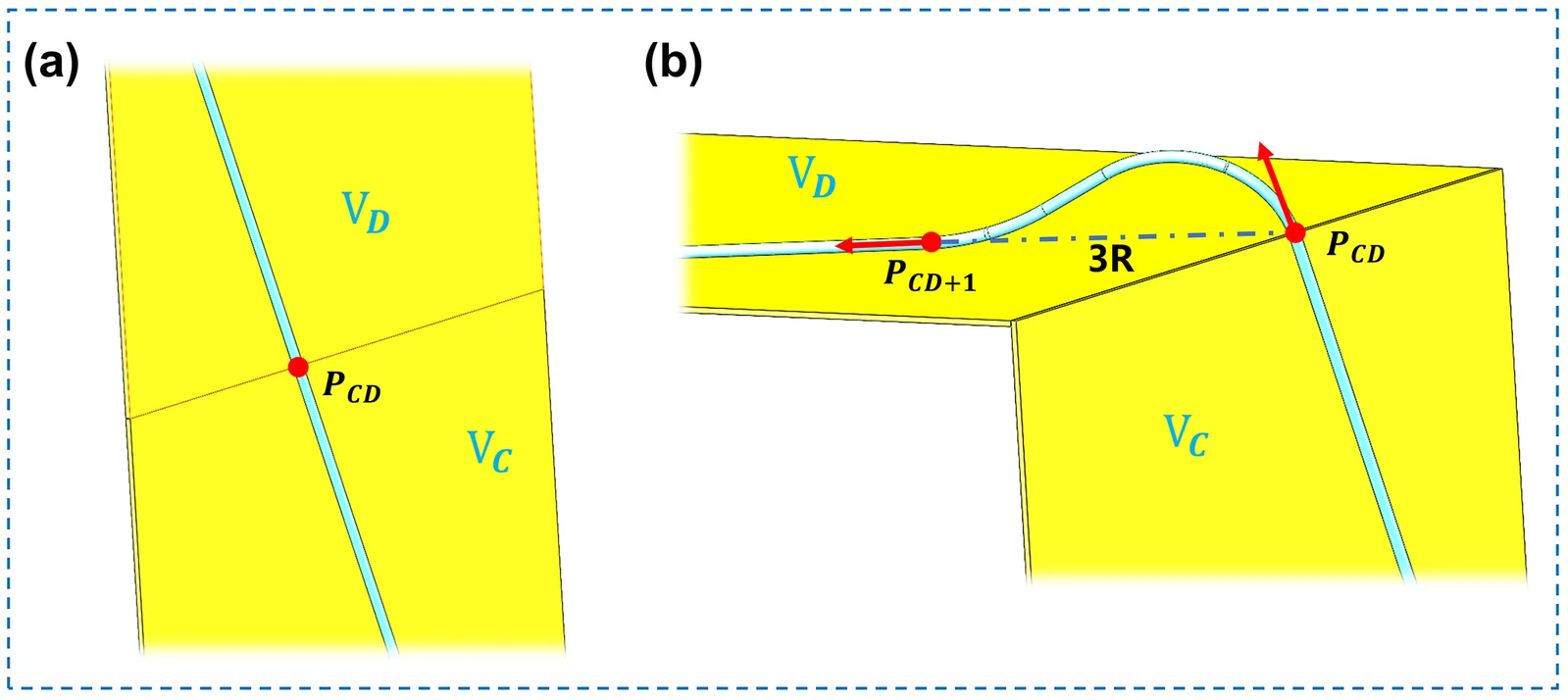
Case 2: (a) 2D Path Planning; (b) Bending treatment.

**Case 3: Non-planar Start/End Points or Direction Vectors.**

If an initial direction vector $P_{s\text{1}}$ lies outside the wall-adjacent plane while the terminal vector $P_{t}$ is within it (Fig 10), a specialized treatment is applied. First, in-plane routing is completed using $P_{s\text{2}}$ (the projection of $P_{s\text{1}}$) and $P_{t}$. A reference circle with radius 3R centered at $P_{s\text{2}}$ identifies an intersection $P_{m}$ on the planar path. Finally, the 3D Dubins engineering transition solver connects $P_{s\text{1}}$ to $P_{m}$, ensuring a feasible localized transition.

**Fig 10:**
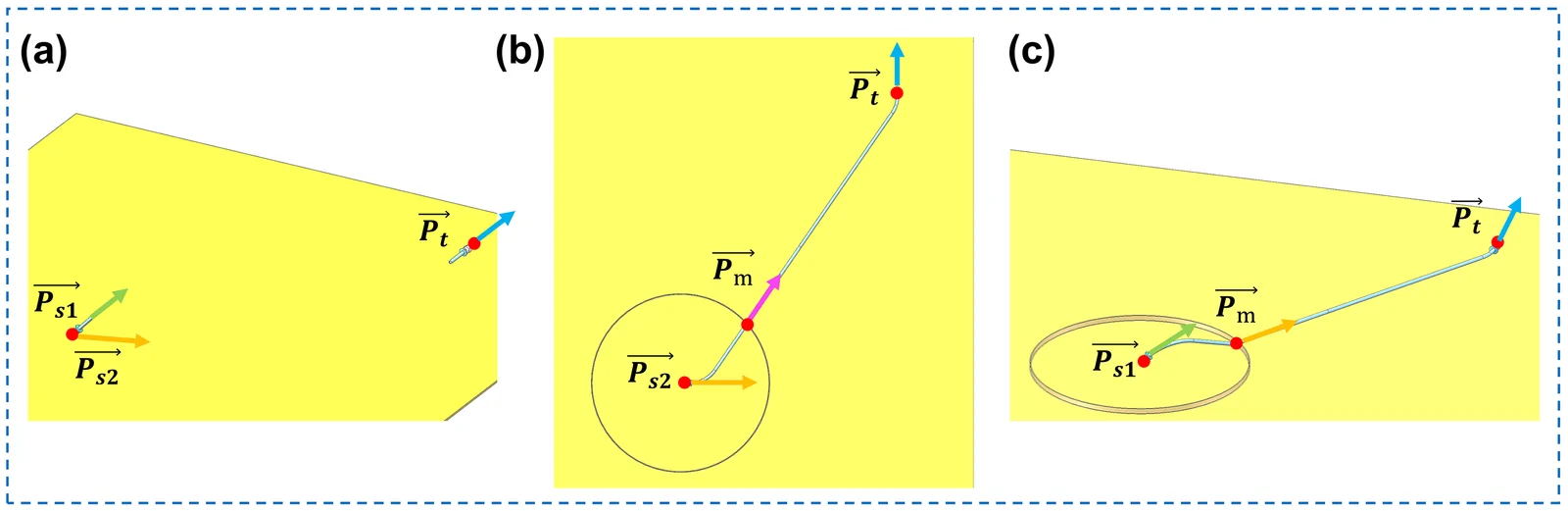
Case 3: (a) Starting point projection; (b) 2D Path Planning; (c) Bending treatment.

**Fig 11:**
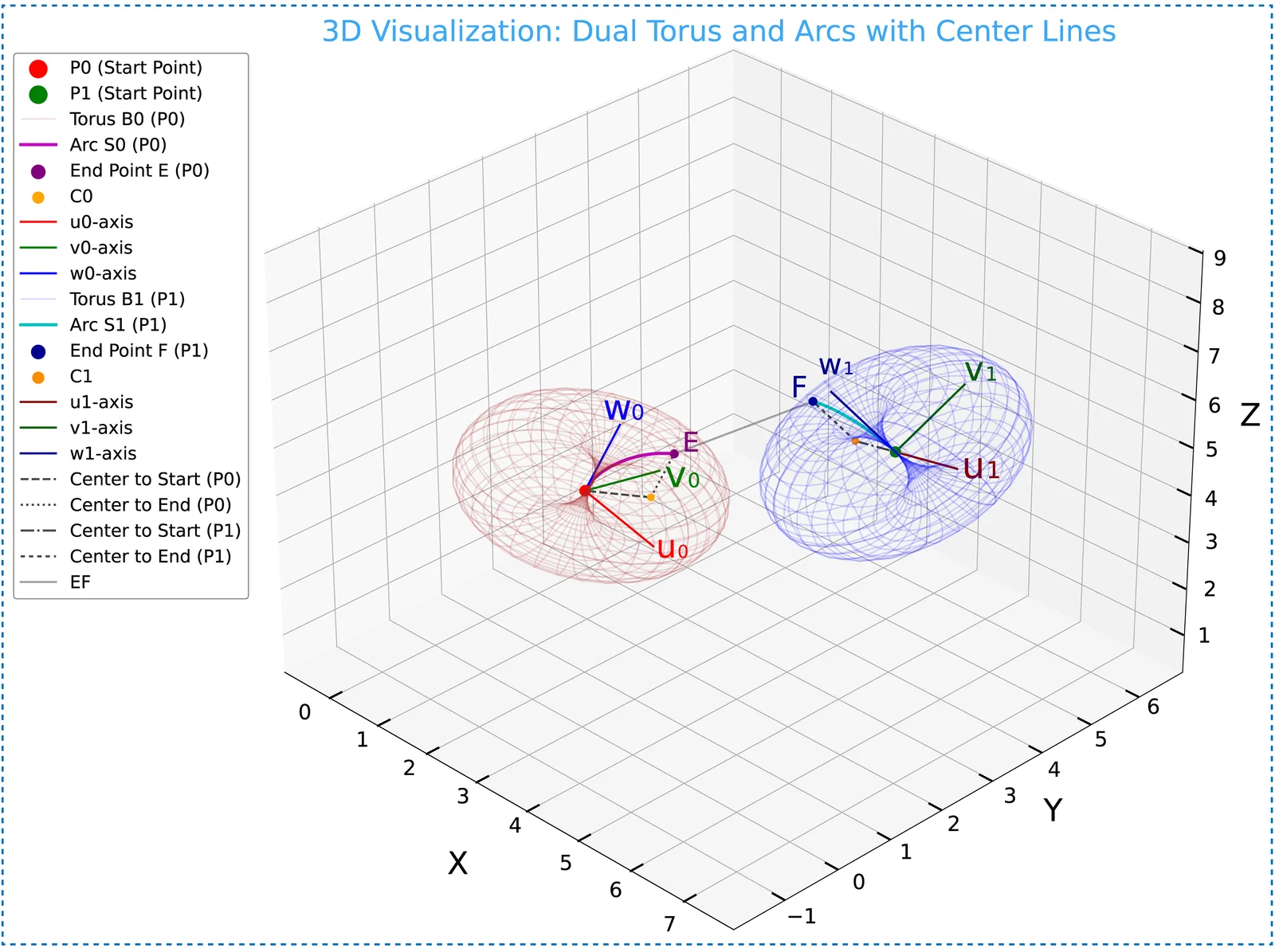
3D. Dubins engineering transition path construction.

#### 3.4.2 3D Dubins engineering transition solver.

Given 3D points $P_{\text{0}}$ and $P_{\text{1}}$ with direction vectors $d_{0}$ and $d_{1}$, the goal is to solve for a 3D Dubins engineering transition path using an arc-straight-arc pattern. This engineering transition solver constructs the curve as a sequence comprising arc $S_{\text{0}}$ (from $P_{\text{0}}$ to E), straight segment $L_{EF}$ (from E to F), and arc $S_{\text{1}}$ (from F to $P_{\text{1}}$). Continuity is maintained such that the initial tangent of $S_{\text{0}}$ aligns with $d_{0}$, and its terminal tangent aligns with $L_{EF}$, as shown in Fig 11. Similarly, the initial tangent of $S_{\text{1}}$ aligns with $L_{FE}$, while its terminal tangent opposes $d_{1}$.

#### 3.4.3 Basic model.

The coordinates for the arc endpoint E and start point F are obtained through the following parametric equations:


$$\begin{array}{c} E(\alpha ,\gamma )=P_{\text{0}}+\left[u_{0},v_{0},w_{0}\right][\begin{array}{c} R\left(\text{1}-\text{cos}\gamma \right)\text{cos}\alpha \\ R\left(\text{1}-\text{cos}\gamma \right)\text{sin}\alpha \\ R\;\text{sin}\gamma \end{array}] \end{array}$$ (21)

$$\begin{array}{c} F(\beta ,\delta )=P_{\text{1}}+\left[u_{1},v_{1},w_{1}\right][\begin{array}{c} R\left(\text{1}-\text{cos}\delta \right)\text{cos}\beta \\ R\left(\text{1}-\text{cos}\delta \right)\text{sin}\beta \\ R\;\text{sin}\delta \end{array}] \end{array}$$ (22)

where α and β denote the rotation angles of arcs $S_{\text{0}}$ and $S_{\text{1}}$ about axes $d_{0}$ and $d_{1}$, while γ and δ represent their respective central angles. The local right-handed orthonormal unit basis $\{u_{i},v_{i},w_{i}\}$ at point $P_{i}$ is derived as:


$$\begin{array}{c} \left\{ \begin{array}{c} @rw_{i}=d_{i}/\left|d_{i}\right|\\ u_{i}=r_{i}-\left(r_{i}\cdot w_{i}\right)w_{i}\\ v_{i}=w_{i}\times u_{i} \end{array}\right. \end{array}$$ (23)

where, $r_{i}$ can be derived as:


$$r_{i}=\{\begin{array}{cc} {}[\text{0},\text{1},\text{0}] & if\;|w_{i}\cdot [\text{1},\text{0},\text{0}]|>\text{0}.\text{9}\\ {}[\text{1},\text{0},\text{0}] & otherwise \end{array}$$ (24)

Based on the computation methods for points *E* and *F* described above, it is easy to obtain the information corresponding to two circular arc segments. Next, the tangential continuity at the junction between each arc and the straight line should be judged. In classical geometry, tangential continuity is assessed by taking the dot product between the tangent vectors at points *E* (or *F*) and the direction vector of the line $L_{EF}$ (or $L_{FE}$), and checking whether this dot product equals 1. Therefore, the minimum error is chosen as the fitness function to seek the coordinate parameters of points *E* and *F* that minimize the tangential continuity error.

Variable: x=(α,β,γ,δ)

Fitness function:


$$\begin{array}{c} \text{min}f_{\text{error}}={\left(\text{1}-T_{E}\cdot L_{EF}\right)}^{\text{2}}+{\left(\text{1}-T_{F}\cdot L_{FE}\right)}^{\text{2}} \end{array}$$ (25)

Constrain conditions: α,β∈[0,2π) and γ,δ∈(0,π]

The unit tangent vectors ***T***_*E*_ and ***T***_*F*_ at points *E* and *F* on the arc segment can be obtained as follows:


$$\begin{array}{c} T_{E}=\left[u_{0},v_{0},w_{0}\right][\begin{array}{c} \text{sin}\gamma \text{cos}\alpha \\ \text{sin}\gamma \text{sin}\alpha \\ \text{cos}\gamma \end{array}] \end{array}$$ (26)

$$\begin{array}{c} T_{F}=\left[u_{1},v_{1},w_{1}\right][\begin{array}{c} \text{sin}\delta \text{cos}\beta \\ \text{sin}\delta \text{sin}\beta \\ \text{cos}\delta \end{array}] \end{array}$$ (27)

The normalized direction vector of the line *EF*, denoted as $\vec{L_{EF}}$, can be expressed as follows:


$$\begin{array}{c} \vec{L_{EF}}=\frac{\text{F}(\beta ,\delta )-\text{E}(\alpha ,\gamma )}{\left\|\text{F}(\beta ,\delta )-\text{E}(\alpha ,\gamma )\right\|}=-\vec{L_{FE}} \end{array}$$ (28)

This study adopts the differential evolution (DE) algorithm to optimize the fitness function. DE uses its global search capability to quickly identify feasible regions in the parameter space and effectively approach a high-quality near-optimal solution, thus completing the optimization process from global exploration to local convergence.

## 4 Results and discussion

### 4.1 Numerical analysis

#### 4.1.1 Generation of a high-quality multi-waypoint Dubins path.

Utilizing the specified start and terminal poses along with eight intermediate waypoints, the PSO algorithm was employed to determine a high-quality feasible multi-waypoint Dubins path under a minimum turning radius constraint ($R_{min}=\text{5})$. The PSO was configured with a population size of 30, a maximum of 100 iterations, and learning factors $c_{\text{1}}=c_{\text{2}}=\text{1}.\text{5}$. The inertia weight ω followed the adaptive strategy described in Eq. (8).

The optimization results are summarized in Table 1 and Fig 12. The total path length converged to a value of 148.76, while the trajectory strictly satisfied the required tangential continuity constraints ($C^{\text{1}}$ continuity). Furthermore, the algorithm exhibited a rapid convergence rate during the iterative process. These findings validate the efficacy of the PSO-based approach in identifying a high-quality multi-waypoint Dubins trajectory within a constrained environment.

**Table 1:** Path generation for many passing points.

Items	P1	P2	P3	P4	P5	P6	P7	P8	P9	P10
Coordinate	(10,5)	(20,15)	(35,20)	(50,15)	(60,25)	(75,30)	(90,20)	(100,10)	(110,25)	(125,15)
Direction	45°	32.76°	358.01°	12.93°	34.79°	0.00°	312.01°	7.83°	16.64°	300°

**Fig 12:**
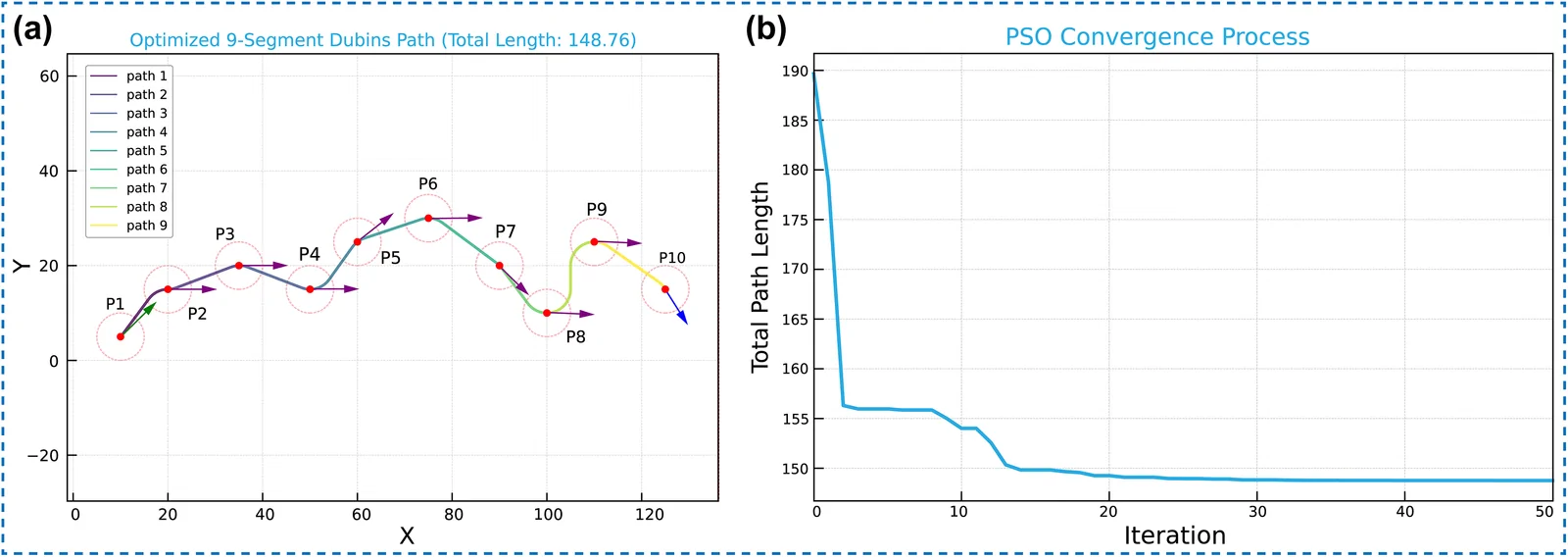
Path generation for many passing points: (a) Final path; (b) Iterative convergence process.

#### 4.1.2 Generation of a 3D Dubins engineering transition path.

To evaluate the performance of the DE algorithm in solving a 3D Dubins engineering transition path, a comparative experiment was conducted against the Nelder-Mead simplex method. Four distinct test cases (specified in Table 2) were utilized, with each algorithm executed independently 30 times per case. To ensure a fair comparison, the population size for DE and the number of initialization restarts for NM were both set to 15. A feasible solution was defined by the optimization error threshold $f_{\text{error}}$. The computational time required for a successful search and the cumulative success rate across 120 total trials were recorded.

**Table 2:** Path generation for non-wall-adjacent points.

Case	$P_{0}$	$d_{0}$	$P_{1}$	$d_{1}$	R	Length	Optimize the error
1	(0,0,0)	(0,0,1)	(5,4,3)	(0,1,0)	1.50	7.70	<1e-6
2	(0,0,0)	(0,0,1)	(5,4,3)	(0,1,0)	2.50	8.26	<1e-6
3	(−1,-2,1)	(0.2,0.5,1)	(4,5,-2)	(−1,-0.3,-1)	1.50	11.25	<1e-6
4	(−1,-2,1)	(0.2,0.5,1)	(4,5,-2)	(−1,-0.3,-1)	2.50	12.82	<1e-6

As illustrated in Fig 13 and Table 3, the DE algorithm demonstrated a significant advantage in computational efficiency. Both the median and mean convergence times were substantially lower than those of the Nelder-Mead algorithm. Furthermore, the success rate convergence curves indicate that DE reached a 100% success rate within approximately 0.28 s. In contrast, Nelder-Mead achieved only a 75.8% success rate after 1.0 s of execution. These results demonstrate that DE possesses enhanced exploration capability and robust convergence performance for the 3D Dubins engineering transition path planning problem.

**Table 3:** Algorithm performance comparison.

Method	DE	Nelder-Mead
Mean time for Case 1	0.185s	0.413s (+123.24%)
Mean time for Case 2	0.217s	0.331s (+52.53%)
Mean time for Case 3	0.181s	0.350s(+93.37%)
Mean time for Case 4	0.199s	0.348s (+74.87%)
Success rate within 1 s	100%	75.8%

**Fig 13:**
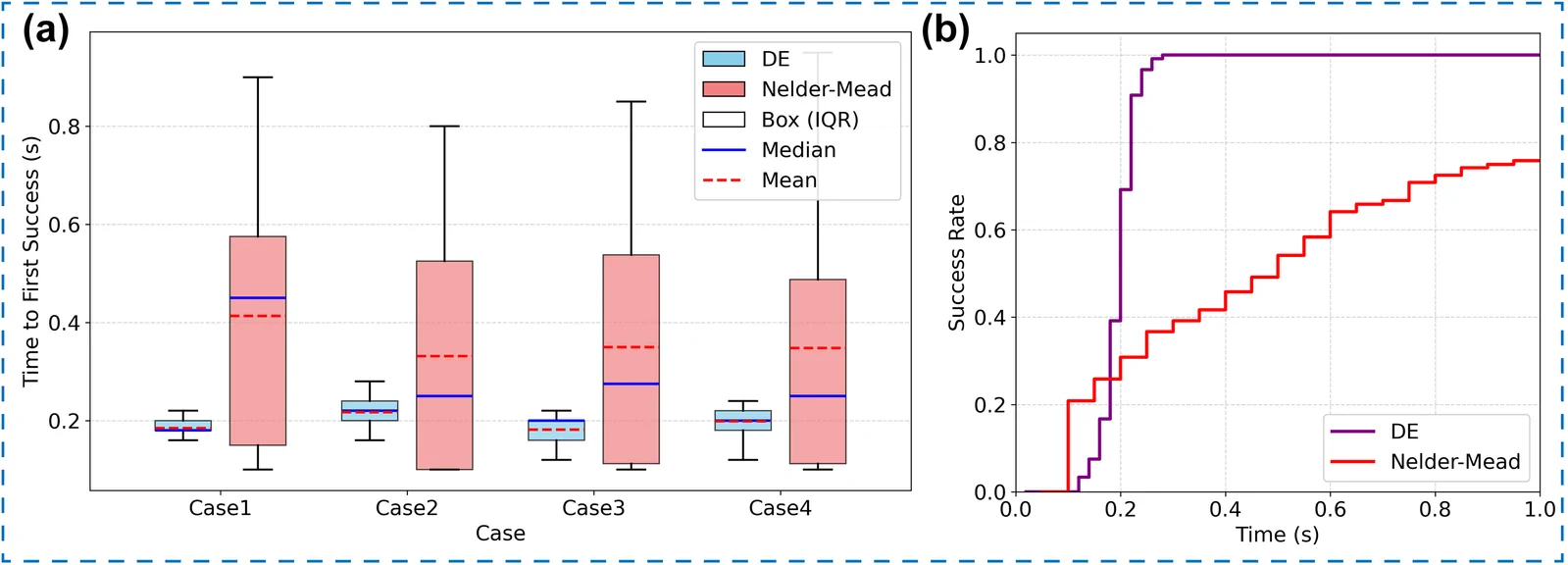
Solution speed analysis: (a) Boxplot of solution times; (b) Success rate convergence.

Representative 3D Dubins engineering transition path solutions for the four test cases are visualized in Fig 14. In Cases 1 and 2, which share the same boundary conditions—start point $P_{\text{0}}$ along direction $d_{0}$ and end point $P_{\text{1}}$ along direction $d_{1}$—the path length increases by 7.3% as the bending radius *R* increases from 1.50 to 2.50. This confirms the theoretical inverse relationship between curvature radius and path compactness. Similarly, in Cases 3 and 4, the path length increases by 14.0% under the same variation of *R*.

**Fig 14:**
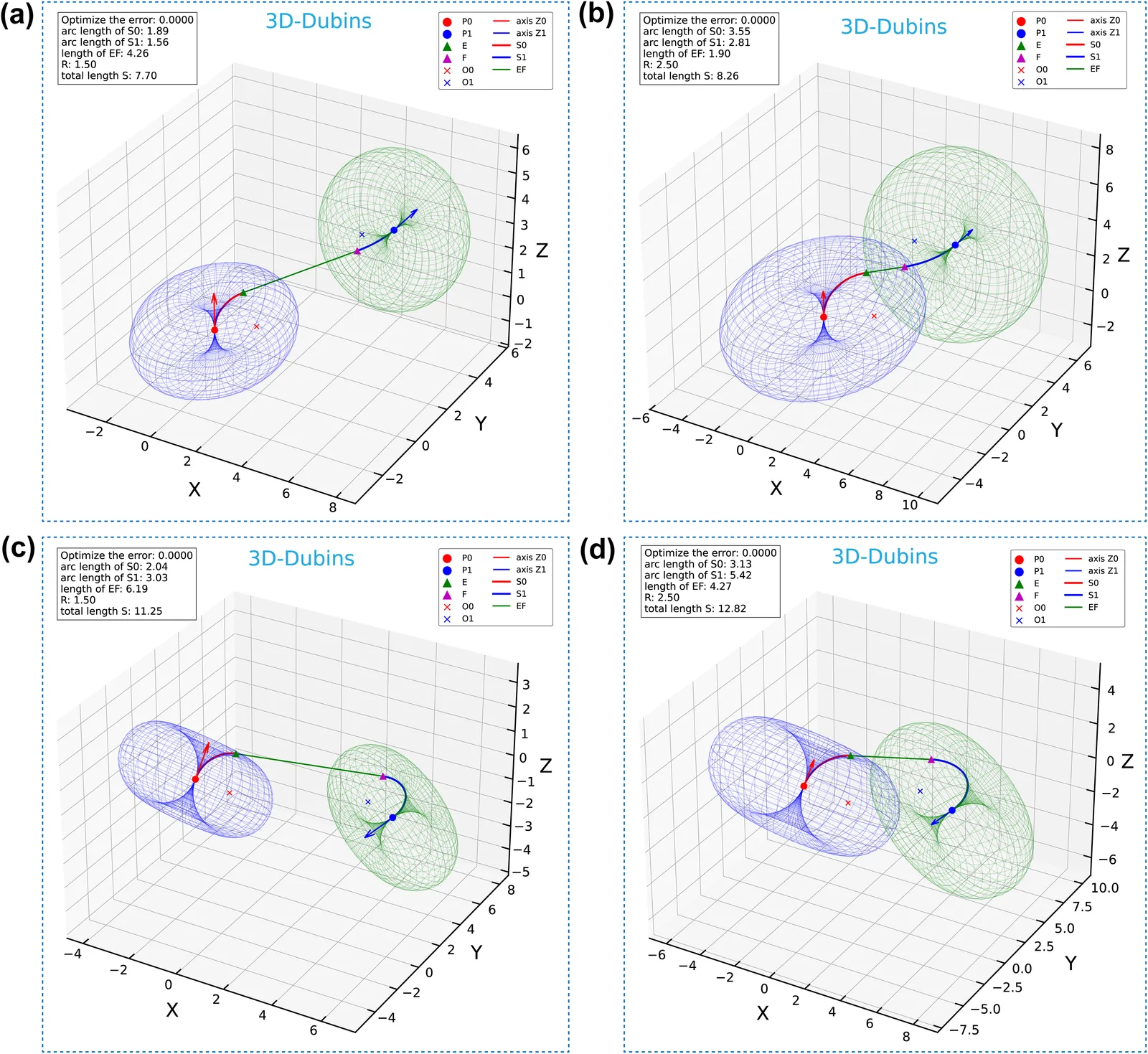
Path generation for non-wall-adjacent points: (a) Case 1; (b) Case 2; (c) Case 3; (d) Case 4.

### 4.2 Validation

#### 4.2.1 Path planning for cables with fixed bending curvature in the plane.

To evaluate the performance of the proposed algorithm under bending curvature constraints within a planar obstacle environment, a comparative simulation was conducted. The simulation workspace was defined as a 400 ×300 mm² area containing six polygonal obstacles. The cable start point S(20,210) and goal point G(390,40) were assigned orientation constraints of ${\text{9}\text{0}}^{\circ }$ and ${\text{2}\text{7}\text{0}}^{\circ }$, respectively.

The proposed method was benchmarked against Hybrid A* and RRT*-Dubins to demonstrate its superiority in handling orientation vectors and curvature constraints. All experiments were executed on a unified hardware platform (Intel Core i9 processor, 16 GB RAM) using the PyCharm Community Edition 2024.1.1 development environment. The specific configurations for the benchmark algorithms were as follows:

Hybrid A*: A classical non-holonomic planning method employing grid-based search and kinematic expansion with a search step of 25 mm.

RRT*-Dubins: An incremental sampling-based method utilizing Dubins curves for node connections with a goal sampling rate of 20%.

All three algorithms utilized a minimum turning radius of 24 mm to enforce curvature constraints. To ensure statistical validity, each experiment was independently repeated 30 times with a 10-s maximum execution time as the termination criterion. Performance was compared based on path length, convergence speed, and success rate.

As shown in Fig 15, (a) illustrates the unconstrained path generated by the basic Dijkstra algorithm without considering direction vector and curvature constraints, which does not meet actual engineering constraint requirements and is shown only for intuitive comparison. Figs 15(b), (c), and (d) depict the median trajectories generated by Hybrid A*, RRT*-Dubins, and the proposed algorithm, respectively. Statistical results are presented in Fig 16: (a) provides a boxplot of path lengths over the 30 trials; (b) illustrates the stepwise convergence for cases corresponding to the median path length; and (c) summarizes the success rates. A comprehensive statistical comparison of all evaluated algorithms is provided in Table 4.

**Table 4:** Algorithm performance comparison.

	Proposed Method	Hybrid A*	RRT*Dubins
Median Path Length	467.83mm	539.11mm	511.36mm
Median time to first path	0.7s	8.4s	0.9s
Median time to final path	4.2s	8.4s	6.4s
Success rate	100%	100%	76.67%
Terminal error	0mm	9.2mm	0mm

**Fig 15:**
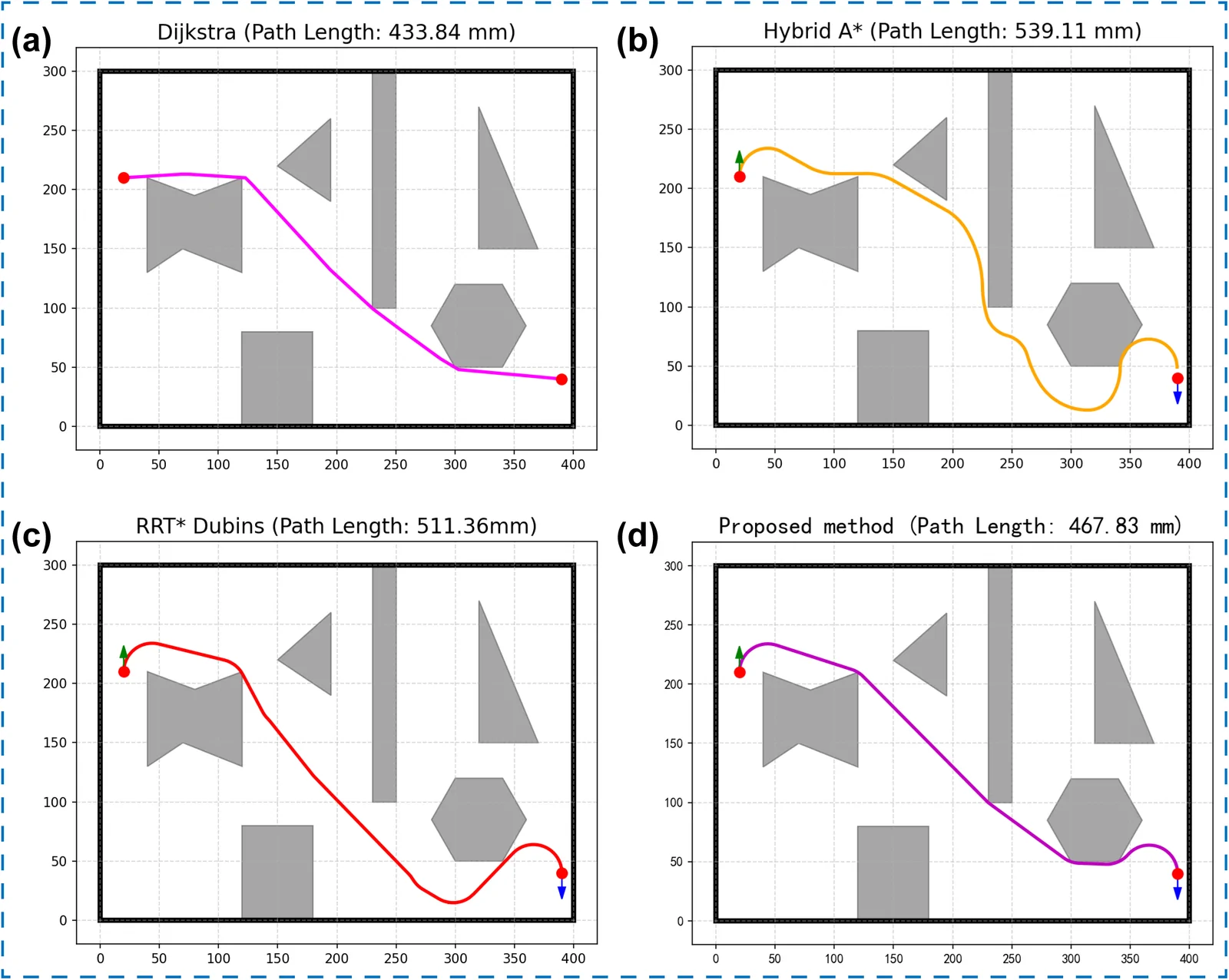
Path length of the median trial: (a) Dijkstra (no curvature/direction constraints); (b) Hybrid A*; (c) RRT*Dubins, (d) Proposed method.

**Fig 16:**
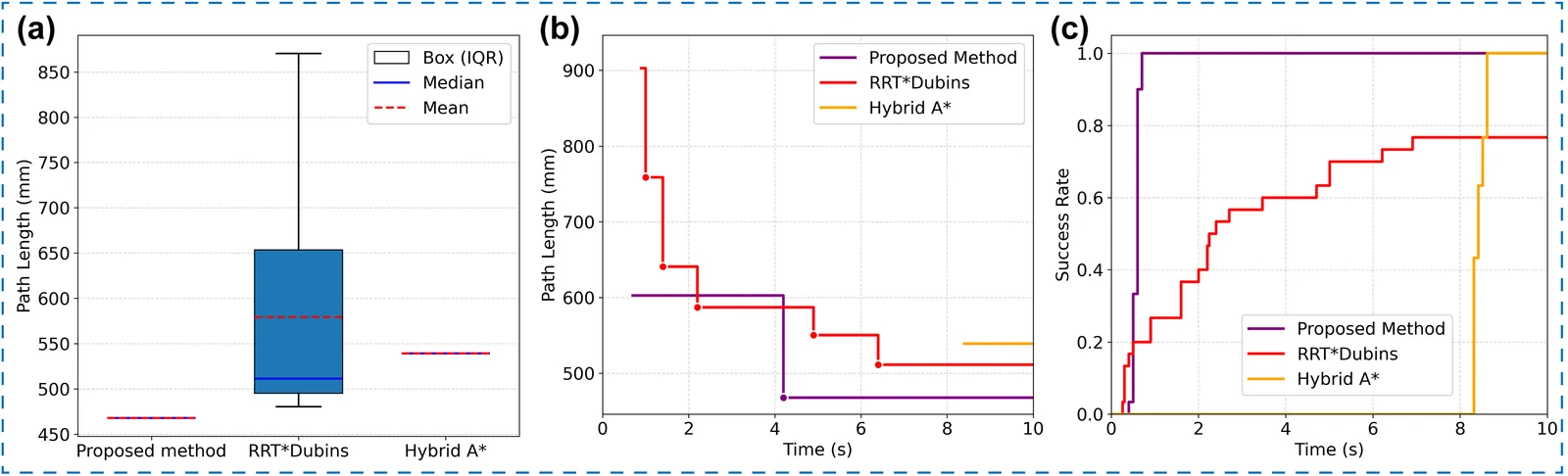
Algorithm performance comparison: (a) Boxplot of path lengths; (b) Stepwise convergence diagram; (c) Path planning success rate.

Experimental comparisons demonstrate that the proposed method outperforms both baseline algorithms across multiple metrics. Compared with Hybrid A*, the proposed method reduces the path length by 13.23% (from 539.11 mm to 467.83 mm), reduces the median time to final path by 50.00% (from 8.4 s to 4.2 s), and reduces the terminal error from 9.2 mm to 0 mm. Compared with RRT*Dubins, the proposed method reduces the path length by 8.51% (from 511.36 mm to 467.83 mm), reduces the median time to final path by 34.38% (from 6.4 s to 4.2 s), increases the success rate by 30.43% (from 76.67% to 100%). Overall, the proposed algorithm searches stably, generates shorter paths, adopts compact detouring strategies in obstacle regions without redundant detours, and better meets the engineering requirements of cable path planning.

#### 4.2.2 Path planning under varying curvature constraints.

To investigate the influence of the minimum bending radius on cable trajectories, the proposed method was executed with radius constraints of 24 mm, 36 mm, and 48 mm while maintaining constant start and terminal poses. The results indicate that the bending radius significantly impacts the trajectory geometry and length. Specifically, the calculated path lengths for the three constraints were 467.83 mm, 498.76 mm, and 521.73 mm, respectively. To validate these theoretical results, physical cables were tailored according to the calculated lengths and installed along the planned paths. The successful alignment between the physical installation and the planned trajectories confirms the practical feasibility of the generated paths (Fig 17).

**Fig 17:**
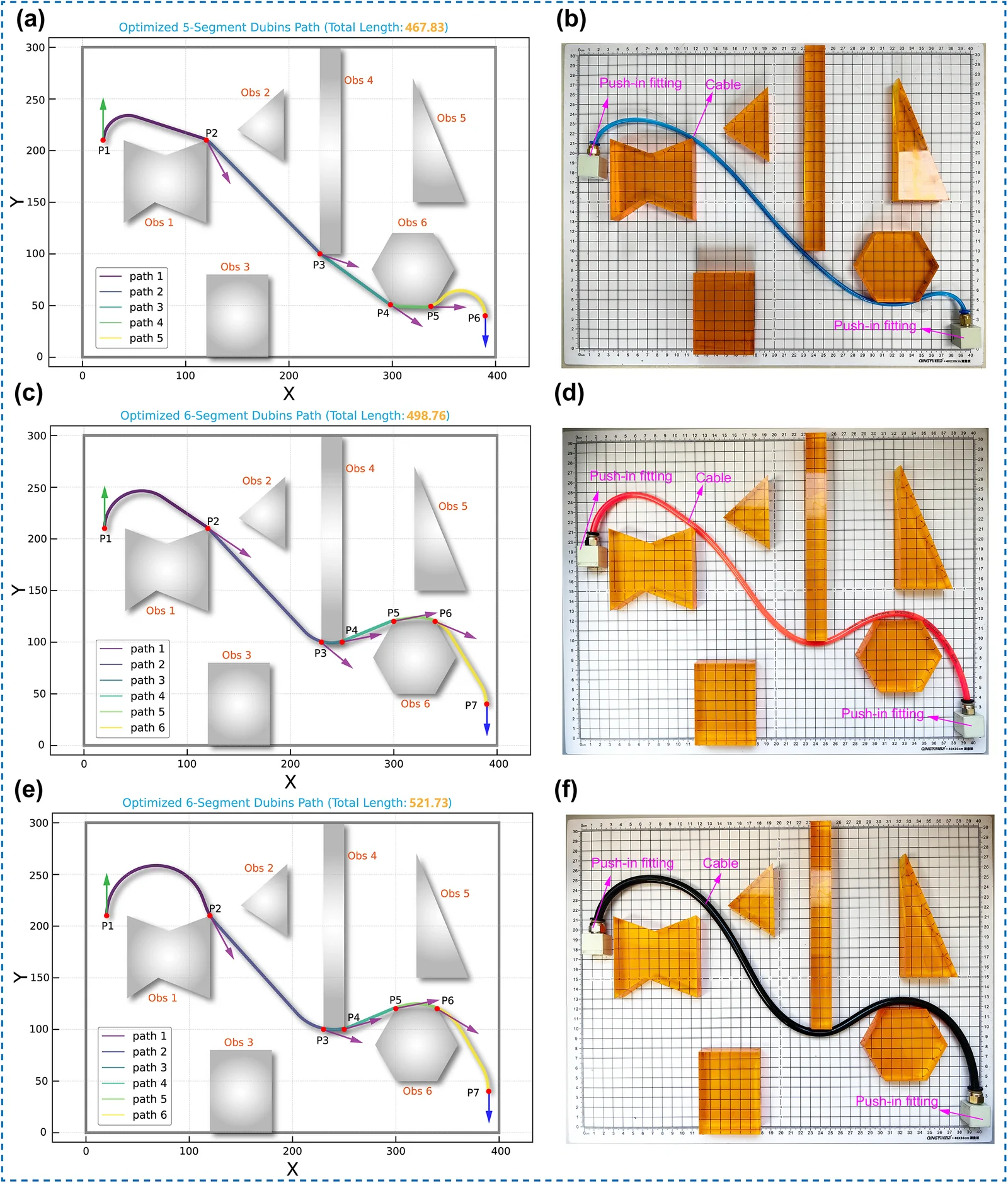
Comparison of cable path planning and experimental validation under varying bending radii: (a) Simulated path with *R* = 24 mm; (b) Physical cable installation with *R* = 24 mm; (c) Simulated path with *R* = 36 mm; (d) Physical cable installation with *R* = 36 mm; (e) Simulated path with *R* = 48 mm; (f) Physical cable installation with *R* = 48 mm.

#### 4.2.3 Out-of-plane start/end points and direction vectors.

Using the same six-obstacle environment, this experiment investigated scenarios where the initial pose (comprising the start point and direction vector) was situated outside the wall-adjacent plane. Following the methodology described in Case 3 of Section 3.3.1, the out-of-plane problem was first projected into a planar equivalent for path planning. Subsequently, a 3D Dubins engineering transition solver was performed to connect the initial out-of-plane segment to the planar waypoint $P_{m}$. A constant bending radius of 24 mm was maintained throughout this process.

Fig 18 illustrates the planning results for this non-coplanar scenario. Physical verification via tailored cable laying demonstrated that the proposed method facilitates a smooth orientation transition at the starting point, effectively accommodating practical installation constraints. Furthermore, the 3D Dubins engineering transition solver successfully resolved the flexibility challenges associated with rope-like structures, yielding a total calculated length of 528.76 mm. The high consistency between the experimental wiring and theoretical planning demonstrates that the proposed post-processing scheme for non-coplanar endpoints satisfies the rigorous requirements of practical engineering.

**Fig 18:**
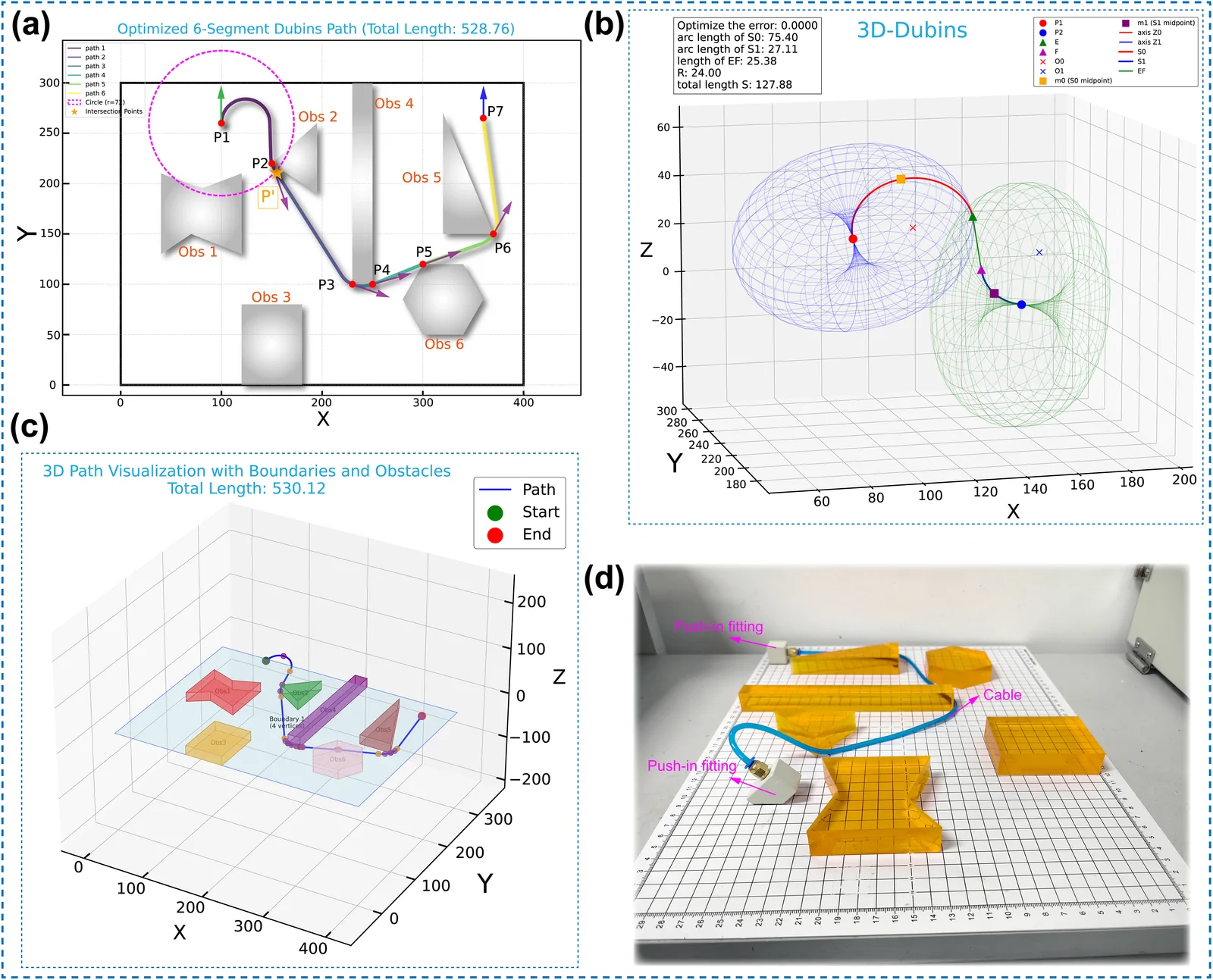
Cable path planning with skew endpoint direction vectors: (a) Path planning under plane projection algorithm; (b) 3D Dubins engineering transition solver; (c) Final theoretical path; (d) Experimental verification.

#### 4.2.4 External bending treatment for adjacent folding planes.

The spatial and cable requirements for a multi-surface environment are depicted in Fig 19. Based on the unfolding methodology described in Section 2, three potential traversal sequences (each involving three surfaces) were identified: ${V\text{2}}_{\text{5}}\to {V\text{2}}_{\text{6}}\to {V\text{2}}_{\text{7}},{V\text{2}}_{\text{5}}\to {V\text{2}}_{\text{4}}\to {V\text{2}}_{\text{7}},and\;{V\text{2}}_{\text{5}}\to {V\text{2}}_{\text{2}}\to {V\text{2}}_{\text{7}}$. Planar trajectories for each sequence were generated using the proposed algorithm, restored to 3D space, and subjected to the bending treatments detailed in Section 3.3.1.

**Fig 19:**
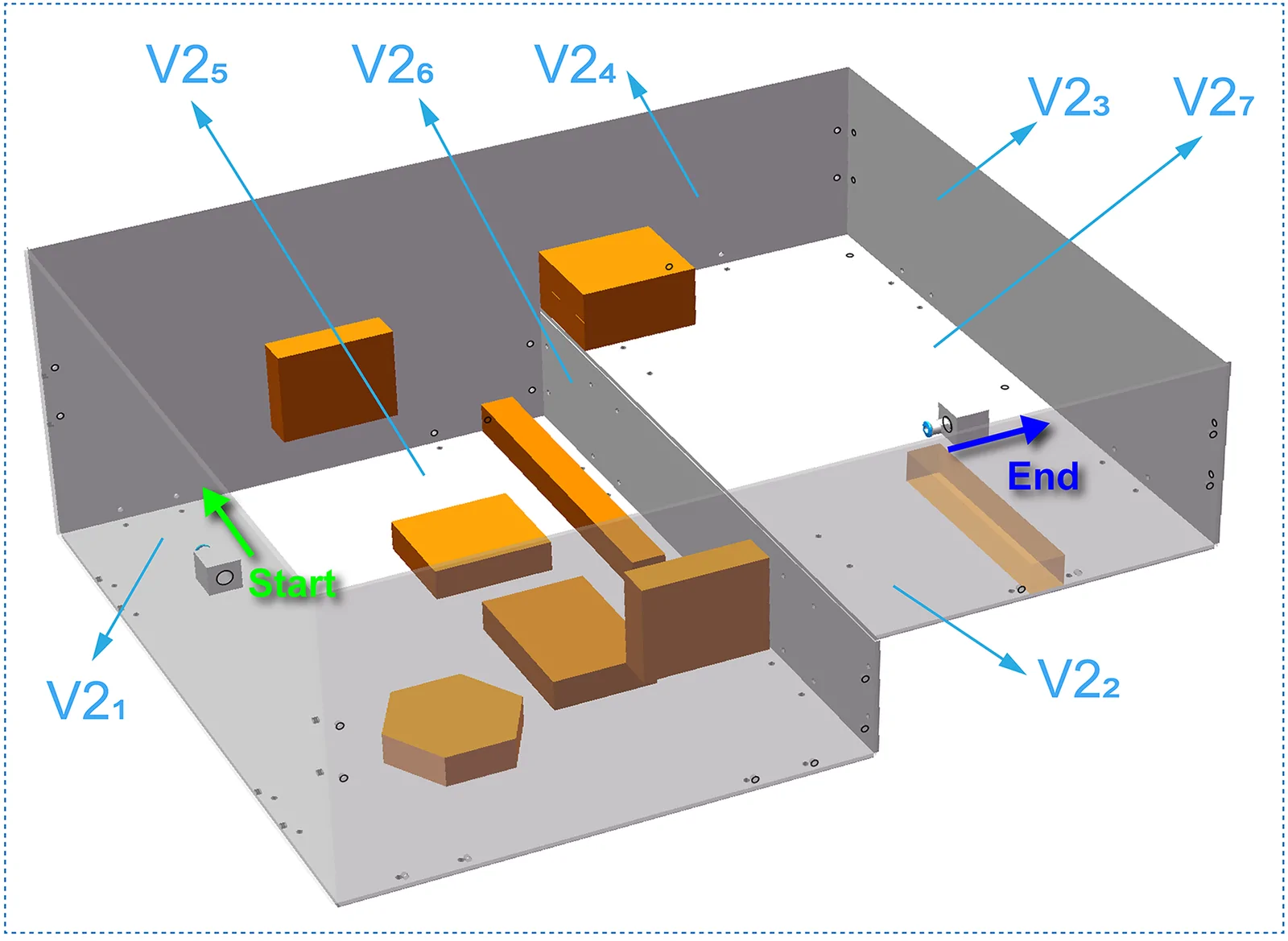
Routing environment.

The resulting spatial trajectories are shown in Fig 20, where the first sequence was identified as the shortest candidate. Following the “planning-before-cutting” forward design approach, cables were cut according to the calculated lengths for all three schemes and installed in the physical environment. Experimental validation confirmed that the proposed method strictly satisfies all engineering constraints, including orientation vectors, bending radii, and wall-adherence requirements. This demonstrates that the integrated framework can significantly streamline on-site installation processes by providing accurate pre-fabrication data.

**Fig 20:**
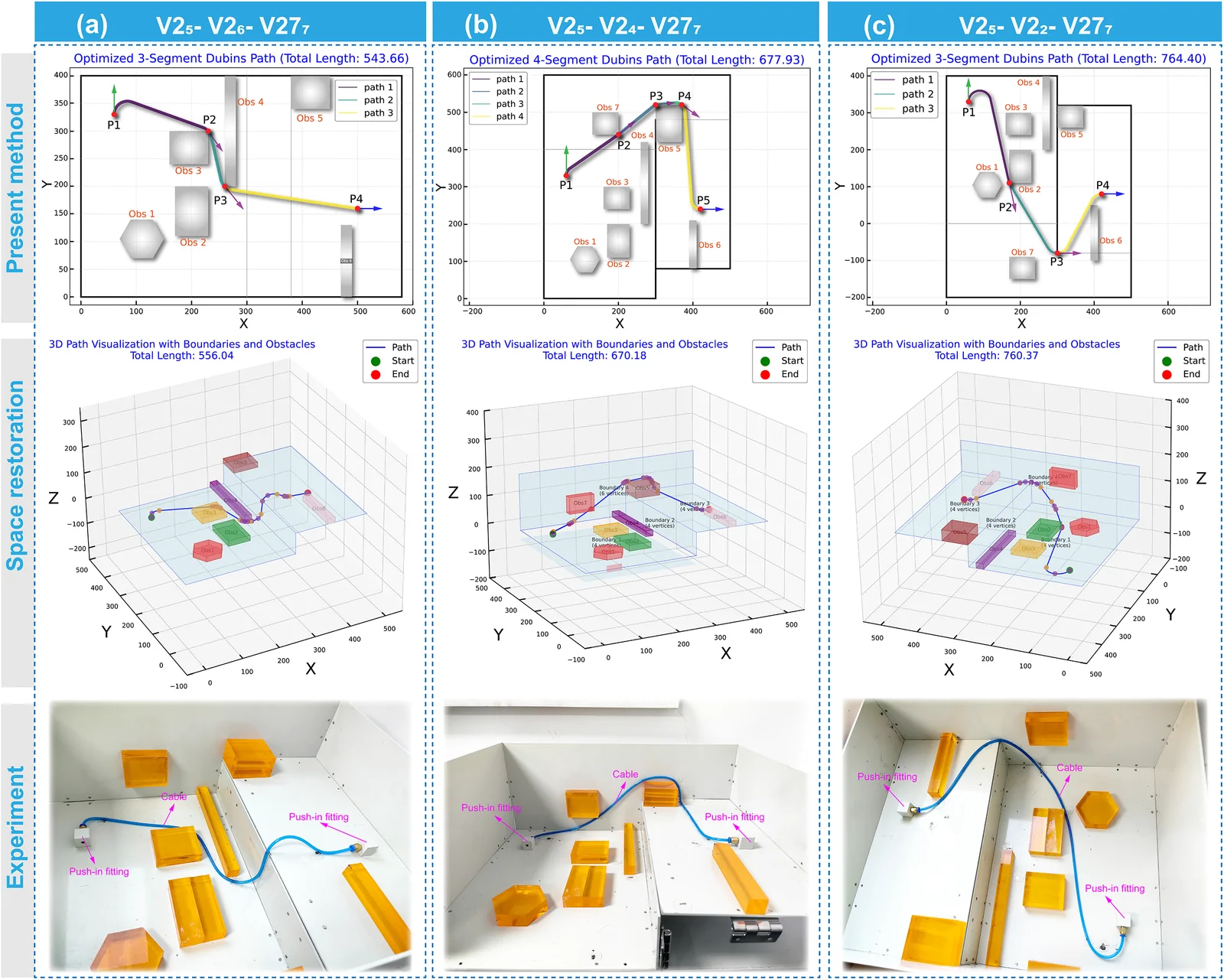
Path planning: (a) First path ${V2}_{5}\to {V2}_{6}\to {V2}_{7}$; (b) Second path ${V2}_{5}\to {V2}_{4}\to {V2}_{7}$; (c) Third path ${V2}_{5}\to {V2}_{2}\to {V2}_{7}$.

## 5 Conclusions

This study addressed the 3D wall-adherent cable routing problem under rigorous orientation and curvature constraints. A hybrid planning framework, designated as IOLPDC, was developed, incorporating a systematic mapping between 3D environmental surfaces and 2D unfolded planes.

The cornerstone of this framework is a local post-insertion strategy that initiates by generating an unconstrained Dubins path, followed by binary-tree-based detours for conflicting obstacles and iterative redundancy pruning. Unlike the traditional “avoidance-before-optimization” paradigm, this approach significantly mitigates computational overhead while preventing the path distortion inherent in conventional post-processing smoothing. Experimental validation within the tested multi-surface environments demonstrated that IOLPDC outperforms Hybrid A* and RRT*Dubins, reducing path lengths by 13.23% and 8.51%, and shortening median planning times by 50.00% and 34.38%, respectively.

The integration of undirected spanning trees and the DE-optimized 3D Dubins engineering transition solver successfully resolved the challenges associated with dimensionality reduction and non-coplanar transitions, ensuring a high-fidelity reconstruction of the cable path throughout the entire 3D-to-2D planning and 3D restoration process.

From an industrial perspective, this research transforms the traditional “cut-to-fit” on-site workflow into a forward design paradigm, enabling precise pre-fabrication and streamlined installation. While the current 3D-to-2D unfolding mechanism is optimized for developable surfaces (e.g., electrical cabinets), its potential for complex pipeline layout planning remains significant.

It should be noted that the 3D-to-2D unfolding mechanism proposed in this study is predicated on the assumption that structural surfaces are developable (e.g., those typical of electrical cabinets) and is currently inapplicable to non-developable geometries, such as hyperbolic panels. Consequently, future research will explore approximate unfolding techniques for such complex surfaces, while simultaneously extending the current 3D Dubins engineering transition solver from the standard “arc-segment-arc” configuration to more sophisticated multi-arc coupled models for intricate environments. Furthermore, to bridge the gap between algorithmic validation and industrial practice, future investigations will transition from simplified polygonal obstacle representations to high-fidelity geometric models, thereby enhancing the overall engineering robustness and applicability of the method.

### Highlights

- A novel local post-insertion strategy (IOLPDC) is proposed to integrate curvature constraints directly into the planning process, avoiding path distortion from traditional smoothing.
- A 3D Dubins engineering transition solver based on differential evolution is presented to handle non-coplanar orientation vectors, ensuring high fidelity between planned and actual cable installation.
- An undirected graph spanning tree method is developed to systematically unfold complex 3D wall-adherent environments into 2D planes for simplified path optimization.

## Supporting information

S1 Table Path generation for many passing points. (PDF)

S2 Table Path generation for non-wall-adjacent points. (PDF)

S3 Table Algorithm performance comparison. (PDF)

S4 Table Algorithm performance comparison. (PDF)

S1 File Minimal data set. (ZIP)
