# Supplementary material for: Spatial layout planning of cable based on a hybrid method combining interference obstacle local post-insertion and improved Dubins curves
Source: PLoS One. 2026 Oct 5;21(10):e0359351. doi: 10.1371/journal.pone.0359351 (PMC13637933; doi:10.1371/journal.pone.0359351)
Supplement: Supplementary file 1 [file pone.0359351.s001.pdf]

Table 1. Path generation for many passing points

Items	P1	P2	P3	P4	P5	P6	P7	P8	P9	P10
**Coordinate**	(10,5)	(20,15)	(35,20)	(50,15)	(60,25)	(75,30)	(90,20)	(100,10)	(110,25)	(125,15)
**Direction**	45°	32.76°	358.01°	12.93°	34.79°	0.00°	312.01°	7.83°	16.64°	300°
