# Supplementary material for: Spatial layout planning of cable based on a hybrid method combining interference obstacle local post-insertion and improved Dubins curves
Source: PLoS One. 2026 Oct 5;21(10):e0359351. doi: 10.1371/journal.pone.0359351 (PMC13637933; doi:10.1371/journal.pone.0359351)
Supplement: Supplementary file 2 [file pone.0359351.s002.pdf]

Table 2. Path generation for non-wall-adjacent points

Case	P_0	d_0	P_1	d_1	R	Length	Optimize the error
**1**	(0,0,0)	(0,0,1)	(5,4,3)	(0,1,0)	1.50	7.70	<1e-6
**2**	(0,0,0)	(0,0,1)	(5,4,3)	(0,1,0)	2.50	8.26	<1e-6
**3**	(-1,-2,1)	(0.2,0.5,1)	(4,5,-2)	(-1,-0.3,-1)	1.50	11.25	<1e-6
**4**	(-1,-2,1)	(0.2,0.5,1)	(4,5,-2)	(-1,-0.3,-1)	2.50	12.82	<1e-6
