# Supplementary material for: Spatial layout planning of cable based on a hybrid method combining interference obstacle local post-insertion and improved Dubins curves
Source: PLoS One. 2026 Oct 5;21(10):e0359351. doi: 10.1371/journal.pone.0359351 (PMC13637933; doi:10.1371/journal.pone.0359351)
Supplement: Supplementary file 3 [file pone.0359351.s003.pdf]

Table 3. Algorithm performance comparison

Method	DE	Nelder-Mead
**Mean time for Case 1**	0.185s	0.413s (+123.24%)
**Mean time for Case 2**	0.217s	0.331s (+52.53%)
**Mean time for Case 3**	0.181s	0.350s(+93.37%)
**Mean time for Case 4**	0.199s	0.348s (+74.87%)
**Success rate within 1 s**	100%	75.8%
