# Supplementary material for: Spatial layout planning of cable based on a hybrid method combining interference obstacle local post-insertion and improved Dubins curves
Source: PLoS One. 2026 Oct 5;21(10):e0359351. doi: 10.1371/journal.pone.0359351 (PMC13637933; doi:10.1371/journal.pone.0359351)
Supplement: Supplementary file 4 [file pone.0359351.s004.pdf]

**Table 4. Algorithm performance comparison**

| | **Proposed Method** | **Hybrid A*** | **RRT*Dubins** |
|---|---|---|---|
| **Median Path Length** | 467.83mm | 539.11mm | 511.36mm |
| **Median time to first path** | 0.7s | 8.4s | 0.9s |
| **Median time to final path** | 4.2s | 8.4s | 6.4s |
| **Success rate** | 100% | 100% | 76.67% |
| **Terminal error** | 0mm | 9.2mm | 0mm |
